# Neurotrophin receptor activation rescues cognitive and synaptic abnormalities caused by hemizygosity of the psychiatric risk gene *Cacna1c*

**DOI:** 10.1038/s41380-020-01001-0

**Published:** 2021-02-17

**Authors:** Cezar M. Tigaret, Tzu-Ching E. Lin, Edward R. Morrell, Lucy Sykes, Anna L. Moon, Michael C. O’Donovan, Michael J. Owen, Lawrence S. Wilkinson, Matthew W. Jones, Kerrie L. Thomas, Jeremy Hall

**Affiliations:** 1 Neuroscience and Mental Health Research Institute, Division of Psychological Medicine and Clinical Neurosciences, School of Medicine, Cardiff University, Cardiff, UK; 2 School of Psychology, Cardiff University, Cardiff, UK; 3 School of Physiology, Pharmacology and Neuroscience, University of Bristol, Bristol, UK; 4 MRC Centre for Neuropsychiatric Genetics and Genomics, Division of Psychological Medicine and Clinical NeurosciencesSchool of Medicine, Cardiff University, Cardiff, UK; 5 School of Bioscience, Cardiff University, Cardiff, UK; 6 Present Address: Neem Biotech, Abertillery, Blaenau Gwent UK

**Keywords:** Neuroscience, Psychiatric disorders

## Abstract

Genetic variation in *CACNA1C*, which encodes the alpha-1 subunit of Ca_V_1.2 L-type voltage-gated calcium channels, is strongly linked to risk for psychiatric disorders including schizophrenia and bipolar disorder. To translate genetics to neurobiological mechanisms and rational therapeutic targets, we investigated the impact of mutations of one copy of *Cacna1c* on rat cognitive, synaptic and circuit phenotypes implicated by patient studies. We show that rats hemizygous for *Cacna1c* harbour marked impairments in learning to disregard non-salient stimuli, a behavioural change previously associated with psychosis. This behavioural deficit is accompanied by dys-coordinated network oscillations during learning, pathway-selective disruption of hippocampal synaptic plasticity, attenuated Ca^2+^ signalling in dendritic spines and decreased signalling through the Extracellular-signal Regulated Kinase (ERK) pathway. Activation of the ERK pathway by a small-molecule agonist of TrkB/TrkC neurotrophin receptors rescued both behavioural and synaptic plasticity deficits in *Cacna1c*^*+/−*^ rats. These results map a route through which genetic variation in *CACNA1C* can disrupt experience-dependent synaptic signalling and circuit activity, culminating in cognitive alterations associated with psychiatric disorders. Our findings highlight targeted activation of neurotrophin signalling pathways with BDNF mimetic drugs as a genetically informed therapeutic approach for rescuing behavioural abnormalities in psychiatric disorder.

## Introduction

The major psychiatric disorders such as schizophrenia and bipolar disorder place an enormous burden on society yet have seen little advances in mechanistic understanding and therapy. While schizophrenia and bipolar disorder can present differently in the clinic, both are associated with psychosis and they have significantly shared genetic architecture [1]. Recent advances in the understanding of the genomic basis of these conditions may pave the way to new therapeutics [2]. Particularly promising in this respect is the demonstration of strong associations of both schizophrenia and bipolar disorder with genetic variations in voltage-gated calcium channels (VGCCs) [3], especially the *CACNA1C* gene, which encodes the pore-forming α_1C_ subunit of Ca_V_1.2 L-type VGCCs (L-VGCCs) [4, 5].

The exact molecular effects of the *CACNA1C* risk-associated single nucleotide polymorphisms (SNPs) are not fully understood. Common risk SNPs in *CACNA1C* are intronic and likely to alter *CACNA1C* gene expression, with decreased expression seen in some, but not all, studies [6–10]. Recent evidence suggests that in human hippocampus the risk SNPs act to reduce *CACNA1C* expression [10]. The association of rare deleterious mutations in genes encoding VGCC subunits, including *CACNA1C*, with schizophrenia and other neurodevelopmental disorders further supports the view that decreased *CACNA1C* expression can contribute to disease risk [11, 12]. Understanding the functional effects of *CACNA1C* dosage, and in particular reduced dosage, is therefore necessary to discerning the contribution of genetic variation in L-VGCCs to neuropsychiatric risk.

Ca_V_1.2 L-VGCCs are highly expressed in the mammalian brain, including hippocampus [13]. Clinical and preclinical studies implicate hippocampal abnormalities in the pathophysiology of schizophrenia and bipolar disorder [14–16], and impaired hippocampus-dependent associative learning in the development of psychosis [17–20]. Hippocampal associative learning is orchestrated by rhythmic neural activity in the entorhinal-hippocampal network [21, 22] and is underpinned by associative synaptic plasticity at glutamatergic synapses, induced by coordinated activation of postsynaptic NMDA receptors (NMDAR) and VGCCs including L-VGCCs [23–26].

At a molecular level L-VGCCs link membrane depolarization to transcription via the Extracellular-signal Regulated Kinase (ERK) signalling pathway which regulates synaptic plasticity [27–30] and activates transcription factors including CREB controlling the expression of genes required for long-term plasticity [29, 30]. Thus, L-VGCC-mediated calcium signalling regulates the changes in synaptic efficacy and gene expression underlying associative learning.

In this study we used a *Cacna1c*^*+/−*^ rat model [31] in order to examine the impact of reduced *Cacna1c* dosage on hippocampal associative learning, synaptic plasticity, circuit activity and ERK signalling. Having mapped the mechanistic links between genetic variation in *Cacna1c* and behavioural phenotype we showed that activation of ERK signalling with a small-molecule TrkB/TrkC neurotrophin receptor agonist could rescue the behavioural and synaptic plasticity impairments.

## Materials and methods

### Animals

*Cacna1c*^*+/−*^ rats [31] with a truncating mutation in exon 6 of *Cacna1c* gene were generated from cryo-preserved embryos (strain SD-*Cacna1c*^*tm1Sage*^, Sage Research Labs, Pennsylvania, USA) and bred at Charles River (Margate, UK), Cardiff and Bristol Universities. We used 278 *Cacna1c*^*+/+*^ and heterozygous littermates, and 54 male Lister Hooded rats (Charles River, UK) for behavioural experiments using intrahippocampal diltiazem (DTZ) infusion. Littermates were housed up to four per cage, with access to food and water *ad libitum*. Experiments were performed on mature animals (age: 12–52 weeks) [32] maintained under normal (12 h/12 h) or reversed light/dark cycle (dark: 10 a.m. to 8 p.m. for behavioural studies; behavioural training at 11 a.m.). Animal handling followed Home Office regulations as directed by Home Office Licensing teams at the host institutions.

### Behavioural, electrophysiological, and molecular methods

These procedures are described in Supplementary Materials and methods.

### Statistical analysis

Sample sizes were determined as described in Supplementary Materials. Animals were assigned pseudo-randomly to experimental groups, with experiments performed blind to genotype. Specific statistical analyses are given in Supplementary Materials and methods. Pooled data are represented as mean ± SEM. Asterisks show statistical significance: *p < 0.05; **p < 0.01; ***p < 0.001.

## Results

### *Cacna1c* hemizygosity disrupts latent inhibition of contextual fear conditioning

**Fig. 1:**
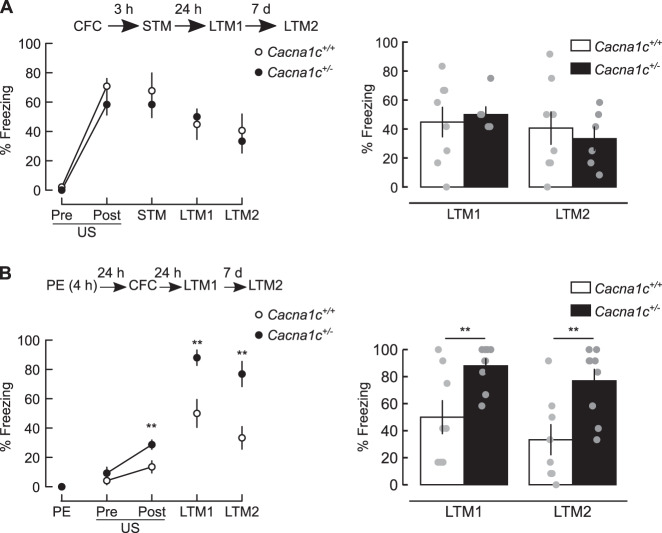
Low *Cacna1c* dosage selectively impairs latent inhibition (LI) of contextual fear memory CFM. All panels: *Left*: schematics of behavioural protocols (*top*) and freezing responses measured during all trials (*bottom*). PE: 4 h context pre-exposure; CFC contextual fear conditioning comprising Pre-US and Post-US epochs before and after footshock (US), respectively; STM, LTM1, LTM2: short-term and long-term contextual fear memory (CFM) retrieval trials. Right: Summary of freezing responses at LTM tests. **A** Acquisition and consolidation of CFC are unaltered in *Cacna1c*^*+/*−^ animals. Effects of genotype: *F*_(1,12)_ = 0.385, *p* = 0.546, trial: *F*_(4,48)_ = 29.624, *p* < 0.001; genotype × trial interaction: *F*_(4,48)_ = 0.533, *p* = 0.712; *Cacna1c*^*+/*−^: *n* = 6; *Cacna1c*^*+/+*^: *n* = 8. **B** Higher levels of freezing post-US and during CFM retrieval trials indicate the *Cacna1c*^*+/−*^ animals have a deficit in the LI. Genotype × session interaction: *F*_(2.1,31.49)_ = 6.119, *p* = 0.005; pairwise comparisons between genotypes: Post-US: *F*_(2.1,31.47)_ = 10.464, *p* = 0.006; LTM1: *F*_(2.1,31.49)_ = 8.895, *p* = 0.009; LTM2: *F*_(2.1,31.49)_ = 9.869, *p* = 0.007 (Greenhouse–Geisser corrected); *Cacna1c*^*+/−*^: *n* = 9; *Cacna1c*^*+/+*^: *n* = 8. Data presented as means ± SEM. **p < 0.01 determined by two-way repeated measures ANOVA followed by pairwise *post-hoc* comparison with Bonferroni correction.

We investigated the effects of reduced Ca_V_1.2 dosage on associative fear learning using rats hemizygous for a truncating mutation in exon 6 of *Cacna1c* gene encoding the pore-forming α_1C_ subunit of Ca_V_1.2 L-VGCCs [33] (“Materials and methods”). *Cacna1c*^+/−^ rats have an approximately 50% decrease in hippocampal *Cacna1c* mRNA and protein [31]. We assessed the behavioural impact of *Cacna1c*^*+/−*^ hemizygosity by testing contextual fear conditioning (CFC) in *Cacna1c*^*+/−*^ and wild-type littermates, a hippocampal-dependent behavioural response that requires L-VGCC activation [34, 35]. During training animals received a single mild footshock (US, 2 s, 0.5 mA) 2 min after being placed in a novel context (Fig. 1A and Supplementary Methods). Context-fear association memory was assessed by measuring the freezing response upon return to the conditioned context 3 h (short-term memory, STM), 24 h (long-term memory, LTM1), and 8 days (LTM2) after CFC training. *Cacna1c*^*+/+*^ and *Cacna1c*^*+/*−^ animals had equivalent levels of fear response throughout training and recall sessions (Figs. 1A and S1), indicating that reduced dosage of *Cacna1c* did not alter contextual fear learning per se.

We next assessed the performance of *Cacna1c*^*+/−*^ and *Cacna1c*^*+/+*^ littermates in a paradigm of latent inhibition (LI) of CFC (schematics in Figs. 1B, S1A). LI is the reduced ability to form conditioned associations to a stimulus (here, the conditioning context) due to pre-exposure to stimulus alone and reflects learning to ignore irrelevant stimuli, which is impaired in psychosis [17, 18, 36]. Animals were pre-exposed to the to-be-conditioned context (PE) for 4 h, then trained for CFC 24 h or 48 h later (Figs. 1B, S1). Pre-exposure produced a robust LI of CFC in *Cacna1c*^*+/+*^ animals, manifested as reduced freezing response at LTM trials, but not in *Cacna1c*^*+/−*^ littermates (Figs. 1B, S1 comparing pre-exposed and non-pre-exposed animals). Therefore, *Cacna1c* hemizygosity selectively impaired LI of CFC. In wild-type animals LI of CFC was disrupted by infusion of L-VGCC antagonist DTZ in dorsal hippocampus during pre-exposure (Fig. S2). Together, these results demonstrate a specific role for hippocampal Ca_V_1.2 channels during the pre-exposure stage in LI of CFC paradigm. We therefore focussed our further physiological studies on the hippocampus.

LI of CFC requires dorsal hippocampal-dependent formation of context-specific memories [37]. We hypothesized that the LI of CFC deficit in *Cacna1c*^*+/−*^ animals reflects a disruption of dorsal hippocampal processes that encode novel environment representations during pre-exposure. Therefore, we investigated the impact of *Cacna1c* hemizygosity on two fundamental hippocampal mechanisms proposed to support memory encoding: associative plasticity at CA1 pyramidal cell synapses [23] and phase–amplitude coupling (PAC) between the theta and gamma oscillations of the local field potential (LFP) in CA1 [21].

### *Cacna1c*^*+/−*^ rats have disrupted synaptic plasticity in dorsal hippocampal CA1

**Fig. 2:**
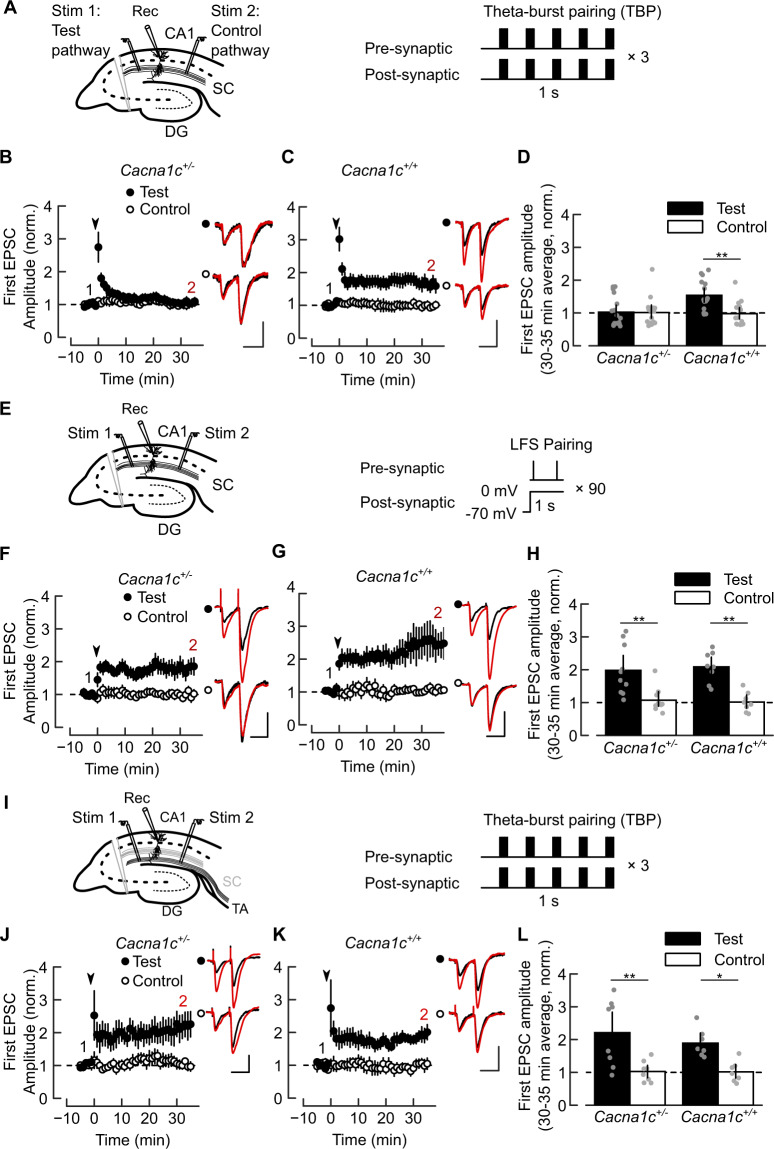
Reduced *Cacna1c* dosage impairs LTCC-sensitive induction of associative LTP selectively at Schaffer collateral to CA1 (SC-CA1) synapses. **A** Electrodes placements (*left*) and theta-burst pairing (TBP) protocol (*right*) for LTP at SC-CA1 synapses. **B** No TBP-induced LTP at SC-CA1 synapses in *Cacna1c*^+/−^ slices. **C** TBP-induced robust homosynaptic LTP at SC-CA1 in *Cacna1c*^+/+^ slices. **D** Summary of normalized change in EPSC amplitude at 30–35 min shown in (**B**) and (**C**). Effect of genotype on LTP: LR_(1)_ = 5.16, *p* = 0.023; genotype × pathway interaction: LR_(1)_ = 7, *p* = 0.0081. Pairwise comparisons of Test vs Control pathway for *Cacna1c*^*+/−*^: *p* = 0.99, *n* = 17 (12: 4 females and 8 males); *Cacna1c*^*+/+*^: *p* = 0.0057, *n* = 16(9). **E** Electrodes placements (*left*) and low-frequency stimulation (LFS-pairing) protocol (*right*) for SC-CA1 LTP. LFS-pairing induces SC-CA1 LTP in *Cacna1c*^*+/−*^ (**F**) and *Cacna1c*^+/+^ slices (**G**). **H** Summary of EPSC amplitude change at 30–35 min shown in (**F**) and (**G**): no effect of genotype (LR_(1)_ = 0.103, *p* = 0.748) or genotype × pathway interaction (LR_(1)_ = 0.227, *p* = 0.633) on LTP. Pairwise Test vs Control for *Cacna1c*^+/−^: *p* = 0.0027, *n* = 10(7); *Cacna1c*^+/+^: *p* = 0.0011, *n* = 8(6). **I** Electrode placements (*left*) and TBP protocol (*right*) for LTP at TA-CA1 synapses. TBP induces homosynaptic TA-CA1 LTP in slices from *Cacna1c*^*+/−*^ (**J**) and *Cacna1c*^*+/+*^ animals (**K**). **L** Summary of changes in normalized EPSC amplitude at 30–35 min shown in (**F**) and (**G**): no effect of genotype (LR_(1)_ = 0.0258, *p* = 0.8724) and no genotype × pathway interaction (LR_(1)_ = 0.065, *p* = 0.798); contrast Test *versus* Control pathways, in *Cacna1c*^*+/−*^: *p* = 0.0093, *n* = 8(7); *Cacna1c*^*+/+*^: *p* = 0.014, *n* = 7(5). Plots show EPSC amplitude time-course in Test and Control pathways, normalized to 5 min average before LTP induction (arrowheads). *Insets*: 5 min average EPSC waveforms before (1, black) and 30–35 min after (2, red) LTP induction; scale bars: 50 pA, 50 ms. Sample sizes given as cells(animals—all males unless indicated). Data presented as means ± SEM. *p < 0.05, **p < 0.01 determined by two-way ordinal regression (cumulative link model) followed by analysis of deviance (ANODE) (colour figure online).

Formation of stable hippocampal context-specific representations requires strengthening of cortical excitatory inputs to CA1 pyramidal neurons via Schaffer collaterals (SC-CA1) from the CA3 area and the temporo-ammonic (TA-CA1) pathway [38–41]. We examined the induction of synaptic long-term potentiation (LTP) at SC-CA1 and TA-CA1 synapses in ex vivo dorsal hippocampal slices from *Cacna1c*^+/−^ and *Cacna1c*^+/+^ rats. We initially used a theta-burst pairing protocol (TBP) consisting of synaptic stimulation coincident with postsynaptic action potentials (APs) (Fig. 2A). TBP mimics theta-burst firing patterns observed in vivo during learning and exploration of novel environments [40, 42, 43] and induces LTP (TBP-LTP) through coordinated activation of postsynaptic NMDAR and VGCCs [26, 44]. TBP failed to induce SC-CA1 LTP in *Cacna1c*^+/−^ animals (Fig. 2B, D). This deficit was present in male and female *Cacna1c*^*+/−*^ animals (Fig. S3), therefore subsequent experiments were performed in males. In *Cacna1c*^+/+^ slices TBP produced robust SC-CA1 LTP (Fig. 2C, D) which was blocked by L-VGCC antagonists isradipine, DTZ, and the NMDAR antagonist L-689560 (Fig. S4A–C, H), confirming NMDAR and L-VGCC requirements for TBP-LTP.

To discern whether non-specific impairments of postsynaptic mechanisms caused the failure of SC-CA1 LTP in *Cacna1c*^+/−^ animals, we tested an alternative LTP induction by pairing low-frequency synaptic stimulation with tonic postsynaptic depolarization (LFS-pairing, Fig. 2E). LFS-pairing generates NMDAR-dependent LTP without postsynaptic APs or L-VGCC contribution [45]. LFS-pairing induced robust SC-CA1 LTP in both genotypes (Fig. 2F–H), which was insensitive to L-VGCC antagonists but was blocked by L-689560 (Fig. S4D–F, H) confirming the dependency of LFS-pairing LTP on NMDARs but not L-VGCCs. In contrast, TA-CA1 synapses exhibited robust TBP-LTP in both genotypes (Fig. 2I–L) which was sensitive to L-VGCC block in wild-type slices (Fig. S4G, H). Paired-pulse facilitation was similar between genotypes (Fig. S5) indicating unaltered neurotransmitter release.

These results show that *Cacna1c* hemizygosity selectively impairs forms of LTP that require L-VGCC activation during postsynaptic AP bursts, without affecting NMDAR-dependent mechanisms, at the SC-CA1 pathway.

### *Cacna1c*^*+/−*^ CA1 pyramidal neurons have impaired spine Ca^2+^ signalling during postsynaptic spike bursts

*Cacna1c*^*+/−*^ CA1 pyramidal neurons had a small reduction in isradipine-sensitive whole-cell calcium currents (Fig. S6) measured using a voltage-ramp method [24] (Supplementary Methods). We hypothesized that the observed neurophysiological deficits arise from local alterations in Ca_V_1.2 availability for processes such as AP repolarization and spine Ca^2+^ signalling, which we investigated next. Neurons from both genotypes had comparable passive membrane properties, AP threshold and rheobase current (Supplementary Table S1, Fig. S7A–E). However, somatic AP broadening during high-frequency (>40 Hz) firing was significantly reduced in *Cacna1c*^+/−^ neurons compared to wild-type cells (Fig. S7F–I). Somatic AP broadening occurs normally during AP bursts, mediated by voltage- and Ca^2+^-sensitive K^+^ channel complexes [46, 47] which can associate Ca_V_1.2 L-VGCCs [48, 49]. In *Cacna1c*^+/+^ neurons isradipine lowered somatic AP broadening to levels comparable to those in *Cacna1c*^+/−^ neurons in the absence of drug (Fig. S7J, K). Therefore, low Ca_V_1.2 dosage appears to alter Ca^2+^-sensitive spike repolarization during burst firing.

**Fig. 3:**
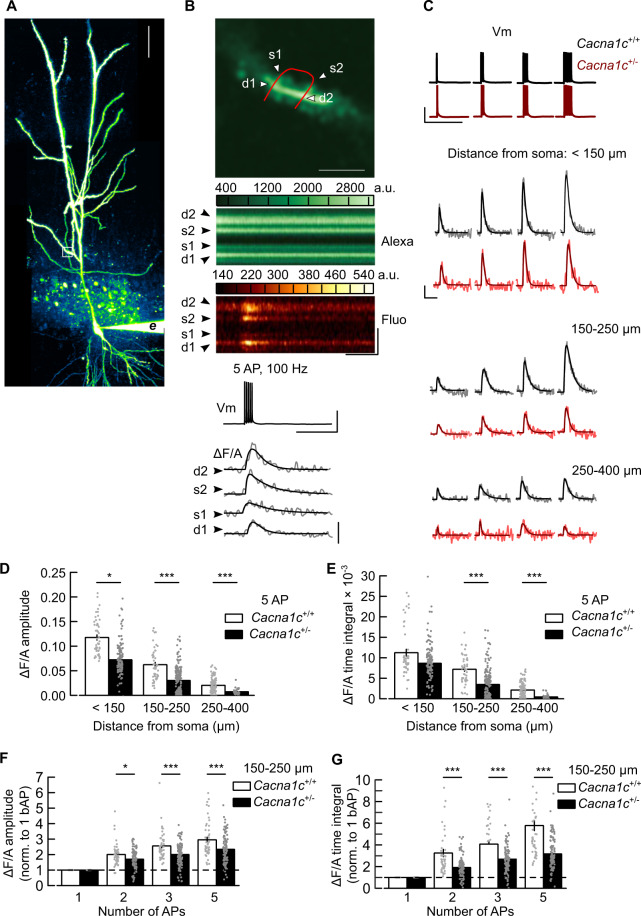
Low *Cacna1c* dosage is correlated with a reduction of spine Ca^2+^ signals elicited by backpropagated action potentials in CA1 pyramidal cells. **A** CA1 pyramidal neuron during spine Ca^2+^ imaging (Z-projected pseudo-colour image, Alexa Fluor 594 channel; *white square*: spine imaging region; *e*: recording electrode; scale: 50 µm). **B** Imaging of spine Ca^2+^ transients elicited by backpropagated action potentials (APCaTs). Top-to-bottom: imaging region in (**A**) with spines (s1, s2), parent dendrite (d1, d2) and line-scan trajectory (red); line-scan images, Alexa Fluor 594 (Alexa) and Fluo 5F (Fluo) channels, during five somatic APs (Vm); APCaTs during the 5 APs (grey: Δ*F*/*A*, change in fluorescence intensity in Fluo channel, relative to Alexa channel; black: double-exponential fit). Scales, vertical: 2 µm, 20 mV, 0.1 arbitrary units (a.u.); horizontal: 0.2 s. **C** Example APCAT waveforms elicited by 1–5 somatic AP (Vm) in spines within three distance zones. Scale: 20 mV, 0.02 a.u., 0.2 s. APCaTs have smaller amplitude (**D**) and time integral (**E**) in *Cacna1c*^*+/−*^ *versus Cacna1c*^*+/+*^ neurons (pairwise comparisons of 5 AP APCaTs, in distance zones (µm) <150: amplitude *p* = 0.0473, time integral *p* = 0.414; 150–250: both measures *p* < 0.0001; 250–400: both measures *p* < 0.0001). **F**, **G** Summation of APCaT amplitude (**F**) and time integral (**G**) with the number of APs is reduced in *Cacna1c*^*+/−*^ *versus Cacna1c*^*+/+*^ neurons (pairwise comparisons for APCaTs at 150–250 µm, for 2 AP: normalized amplitude *p* = 0.15, normalized time integral *p* < 0.0001, 3 AP: both measures *p* < 0.0001; 5 AP: both measures *p* < 0.0001). Data were expressed as means ± SEM. *p < 0.05, **p < 0.01 and ***p < 0.001 determined by two-way repeated measures ANOVA followed by Tukey adjustment of *p* values for contrasts. Statistical analysis results and sample sizes are given in Supplementary Table S2 and Fig. S8; all *p* values for pairwise comparisons are given in Supplementary Table S3 (colour figure online).

AP broadening in CA1 pyramidal neurons is thought to facilitate dendritic backpropagation of somatic spikes [50] necessary for associative synaptic plasticity such as TBP-LTP [26, 44] and amplification of Ca^2+^ signals during AP bursts [47]. Ca_V_1.2 L-VGCCs are expressed in dendrites and dendritic spines [51, 52], and are activated by backpropagated APs [26, 51]. The impaired AP broadening during burst firing in *Cacna1c*^+/−^ neurons may impact plasticity induction by altering dendritic spine Ca^2+^ signals triggered by backpropagated APs. We tested this hypothesis by analyzing Ca^2+^ transients elicited with AP bursts (APCaTs) in spines of radial oblique dendrites (Figs. 3A, B and S8, Supplementary Tables S2 and S3). APCaTs attenuated with increasing distance from soma (Figs. 3C–E and S9). Compared to wild-type cells, the APCaTs in spines at 150–250 µm from soma in *Cacna1c*^+/−^ neurons were consistently smaller (Fig. 3D, E) and scaled poorly with AP burst size (Figs. 3F, G and S10).

These results show that low *Cacna1c* dosage in CA1 pyramidal neurons alters AP-associated spine Ca^2+^ signalling at SC-CA1 synapses, which may underlie the observed selective deficit in synaptic plasticity.

### *Cacna1c*^*+/−*^ animals have reduced Phase-Amplitude Coupling between theta and gamma oscillations of dorsal CA1 LFP

The establishment of neuronal ensembles involved in novel environment encoding depends on the synchronization of excitatory inputs in the CA1 subfield, facilitating synaptic plasticity [21, 53, 54]. To determine the impact of *Cacna1c* heterozygosity on dorsal CA1 network synchronization, we monitored the modulation of LFP gamma oscillations by the phase of LFP theta oscillations (theta–gamma Phase-Amplitude Coupling, PAC). Hippocampal theta–gamma PAC is a proposed mechanism for storage and recall of object and event representations [22, 55, 56]. The slow (~25–40 Hz) and fast (~ 65–140 Hz) gamma oscillations in dorsal CA1 reflect the synchronization between pyramidal neurons in CA1 with neurons in CA3 (via SC-CA1 pathway) and entorhinal cortex (EC) (via TA-CA1 pathway) [57].

**Fig. 4:**
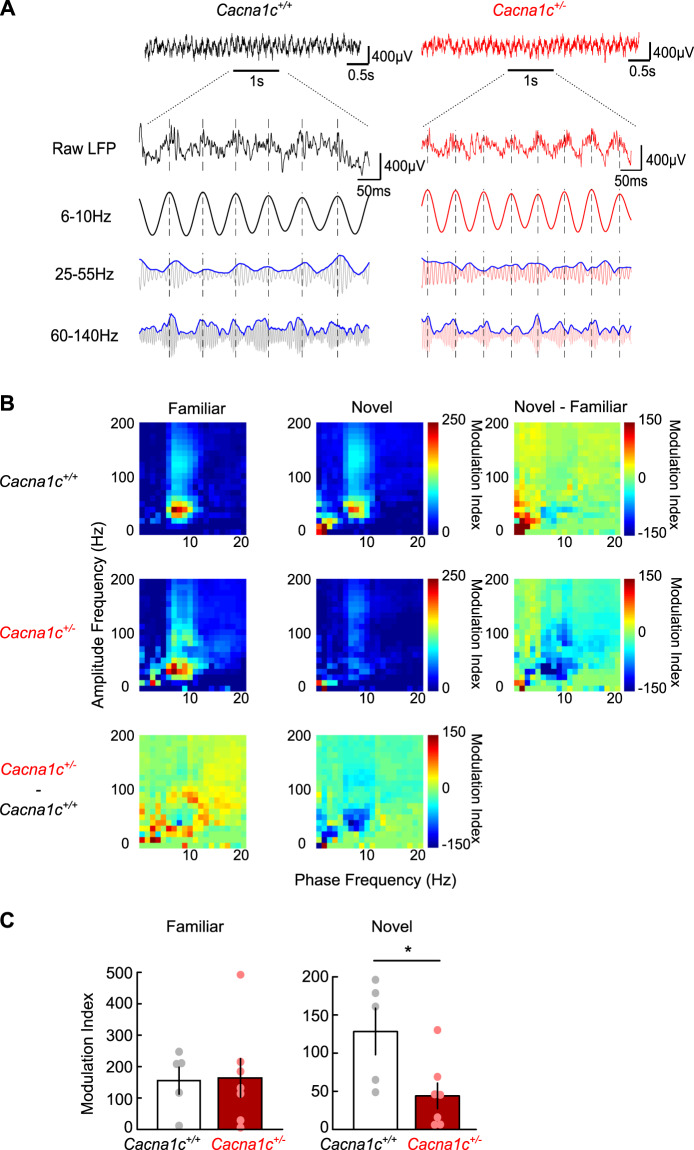
Reduced theta-slow-gamma coupling in a novel environment in *Cacna1c*^+/−^ rats. **A** Local field potentials taken from runs on the familiar track. *Top*: Representative broadband LFP traces (0.1–475 Hz) taken from dorsal CA1 in a 5 s window while running. *Bottom*: 1 s expanded segment showing raw unfiltered LFP (*top*) and bandpass filtered theta (6–10 Hz), slow gamma (25–55 Hz) and fast gamma (60–140 Hz) (*bottom*). Phase-amplitude coupling measures the degree to which the amplitude envelope (blue traces shown for slow and fast gamma) is modulated by the phase signal (theta filtered LFP). Black: *Cacna1c*^*+/+*^; red: *Cacnac1*^*+/−*^; dashed vertical lines mark timings of theta cycle peaks. **B** Mean phase–amplitude coupling comodulograms taken from track runs on the familiar and novel track. Colour represents the modulation index (MI) depicting the degree of coupling between the phase of frequencies (1–20 Hz) on the *x*-axis and the amplitude of frequencies (1–200 Hz) on the *y*-axis. Differences between environments and genotypes are shown next to the mean plots as labelled (*Cacna1c*^*+/+*^: *n* = 5; *Cacna1c*^*+/−*^: *n* = 7). **C** Mean phase–amplitude coupling between theta (6–10 Hz) and slow gamma (25–55 Hz). Theta–slow gamma coupling was lower in *Cacna1c*^*+/*−^ rats than *Cacna1c*^*+/+*^ rats on the novel track (*p* = 0.0256) but not on the familiar track (*p* = 0.9184). *Cacna1c*^*+/+*^: *n* = 5; *Cacna1c*^*+/−*^: *n* = 7; Data presented as means ± SEM. *p < 0.05, Student’s *t* test (colour figure online).

To determine the changes in theta–gamma PAC during the exploration of a novel environment we recorded dorsal CA1 LFP oscillations in rats running along a track in a familiar, then a novel environment (Figs. 4A and S11A–C). This approach accounts for correlations of hippocampal theta and gamma rhythms and their coupling with movement [58–60]. In the familiar environment, animals from both genotypes had similar PAC across the gamma frequency spectrum (Fig. 4B, C, “Familiar” sub-panels) and power spectra of LFP oscillations in the 1–40 Hz range (Fig. S11D, E). Upon switching to novel environment, the PAC levels in *Cacna1c*^+/+^ were similar to the initial response to the subsequently familiar environment (Fig. 4B, C, “Novel” sub-panels). *Cacna1c*^*+/*−^ animals had significantly impaired theta–gamma PAC response (Fig. 4B, C) in the novel environment, unrelated to behavioural response to novelty (Fig. S12). Our results reveal a CA3–CA1 network mis-coupling in *Cacna1c*^*+/−*^ animals, which may further disrupt hippocampal encoding of contextual information and contribute to the observed LI deficit.

### A TrkB/TrkC agonist rescues synaptic plasticity and behavioural deficits in *Cacna1c*^*+/−*^ animals

**Fig. 5:**
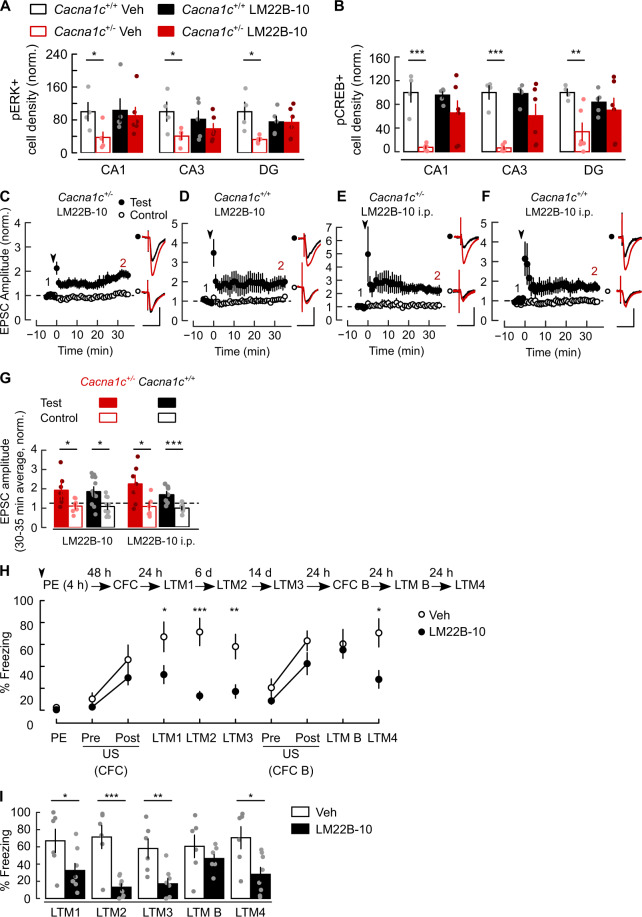
The TrkB/TrkC receptor agonist LM22B-10 rescued dorsal hippocampal ERK-CREB signalling, synaptic plasticity, and LI of CFC in *Cacna1c*^+/−^ rats. Systemic administration of the TrkB/TrkC agonist LM22B-10 (25 mg/kg, i.p.) restored the baseline levels of pERK (**A**) and pCREB (**B**) in dorsal hippocampus of *Cacna1c*^*+/−*^ rats; pERK genotype × treatment: *F*_(1,16)_ = 4.567, *p* = 0.048; genotype effect, vehicle-treated (Veh): *F*_(1,16)_ = 12.866, *p* = 0.002; LM22B-10-treated: *F*_(1,16)_ = 0.615, *p* = 0.444; pCREB genotype × treatment: *F*_(1,17)_ = 5.239, *p* = 0.035; genotype effect, Veh: *F*_(1,16)_ = 21.319, *p* = 0.000, and LM22B-10: *F*_(1,16)_ = 2.485, *p* = 0.133. There was no significant treatment effect in *Cacna1c*^*+/+*^ animals (pERK: F_(1,21)_ = 0.54, *p* = 0.47; pCREB: *F*_(1,21)_ = 0.98, *p* = 0.33). Bars represent summary of immunopositive cell densities in CA1, CA3 and dentate gyrus (DG) of dorsal hippocampus, normalized to average *Cacna1c*^*+/+*^ values. Examples of immunoreactive sections are shown in Fig. S14. Animals were sacrificed 60 min after i.p. administration of drug or vehicle and processed for both ERK and CREB; sample sizes for *Cacna1c*^*+/+*^: LM22B-10 *n* = 5, Veh *n* = 4; *Cacna1c*^*+/−*^: LM22B-10 *n* = 6, Veh *n* = 5 (pERK) and *n* = 6 (pCREB). **C–G** LM22B-10 rescued SC-CA1 LTP induction with TBP in slices from *Cacna1c*^*+/−*^ rats, either during bath application (2 µM, C) or after i.p. bolus (25 mg/kg, E). LM22B-10 had no effect on TBP-induced SC-CA1 LTP in wild-type slices, either during bath application (**D**) or after i.p. bolus (**F**). Insets: 5 min average EPSC waveforms before (1, black) and 30–35 min after (2, red) LTP induction; scale bars: 50 pA, 50 ms. **G** Summary of change in normalized mean EPSC amplitude at 30–35 min after LTP in (**C**–**F**). Pairwise test vs control, **C** *p* = 0.034, *n* = 12(4); **D** *p* = 0.032, *n* = 7(4); **E** *p* = 0.044, *n* = 7(4); **F** *p* = 0.008, *n* = 7(5); sample sizes given as cells(animals). **H** Intrahippocampal infusion of LM22B-10 before context pre-exposure rescued LI of CFC in *Cacna1c*^*+/−*^ animals. *Schematic*: behavioural paradigm in Fig. S1 extended with memory retrieval trials in LI context (LTM3, LTM4), alternative context training (CFC B) and retrieval (LTM B). *Arrowhead*: bilateral infusion of 1 µl LM22B-10 (2 µM, *n* = 8) or vehicle (Veh, *n* = 6) in dorsal hippocampus 60 min before PE. The plot shows the freezing response in all sessions. **I** Summary of freezing response at LTM trials. The freezing responses in LM22B-10 and Veh groups were different (treatment × stage: *F*_(5,60)_ = 5.205, *p* = 0.002). Compared to the vehicle group, LM22B-10 animals showed reduced freezing responses only in the LI context (between-subject contrasts *vs* Veh, LTM1: *F*_(1,11)_ = 4.162, *p* = 0.033, LTM2: *F*_(1,11)_ = 18.467, *p* = 0.0005, LTM3: *F*_(1,11)_ = 9.602, *p* = 0.005, LTM4: F_(1,11)_ = 6.886, *p* = 0.012, LTM B: *F*_*(*1,11)_ = 0.138, *p* = 0.358). All data are presented as means ± SEM. *p < 0.05, **p < 0.01 and ***p < 0.001, two-way repeated measures ANOVA then pairwise *post-hoc* comparison with Bonferroni correction (**A**, **B**, **G**, **H**) and two-way ordinal regression (ANODE) then Tukey-adjusted *post-hoc* comparisons (colour figure online).

We next investigated molecular changes associated with altered hippocampal synaptic plasticity and network activity in *Cacna1c*^*+/*−^ animals. The deficit in AP-elicited spine Ca^2+^ signals in *Cacna1c*^*+/*−^ rats may impact on Ca^2+^-dependent ERK signalling, which couples Ca_V_1.2 L-VGCCs activation to synaptic plasticity [27, 28] and CREB-regulated gene expression for long-term memory [29, 30]. Phosphorylated ERK (pERK, Fig. 5A) and CREB (pCREB, Fig. 5B) immunoreactivities were significantly reduced in *Cacna1c*^*+/−*^ dorsal hippocampus. A separate cohort replicated these findings (Fig. S13A, C) without genotype effect on total ERK and CREB levels (Fig. S13B, D). We detected no differences in hippocampal morphology and cellular density between genotypes (Fig. S14). These results indicate impaired ERK- and CREB-mediated synapse-to-nucleus signalling in *Cacna1c*^+/−^ rats, that may contribute to the observed behavioural and hippocampal dysfunctions. We tested this hypothesis by directly activating ERK signalling using a recently characterized small-molecule TrkB/TrkC neurotrophin receptor co-activator LM22B-10 with brain-derived neurotrophic factor (BDNF) mimetic properties including ERK activation in neurons [61]. Systemic administration of LM22B-10 in *Cacna1c*^+/−^ rats (25 mg/kg i.p., yielding micromolar concentrations in the brain [61]) restored the baseline levels of pERK and pCREB 60 min after injection (Figs. 5A, B and S15) without significant effect in wild-type littermates. We next determined whether LM22B-10 could rescue hippocampal synaptic plasticity in *Cacna1c*^+/−^ rats. LM22B-10 rescued TBP-LTP at SC-CA1 synapses in *Cacna1c*^+/−^ slices, either during bath-application in vitro (Fig. 5C, G) or in slices prepared 60 min after intra-peritoneal administration (Fig. 5E, G), without effect in *Cacna1c*^*+/+*^ littermates (Fig. 5D, F, G). Thus, activation of TrkB/TrkC receptors with local or systemic administration of a BDNF mimetic drug bypasses Ca_V_1.2 L-type VGCC signalling to rescue ERK signalling and associative hippocampal plasticity in *Cacna1c*^+/−^ rats.

The reversal of molecular and synaptic plasticity deficits in *Cacna1c*^+/−^ rats with LM22B-10 prompted us to investigate whether activation of hippocampal TrkB/TrkC receptors could rescue the observed deficit in LI of CFC. A single intra-dorsal hippocampal administration of LM22B-10 60 min before the pre-exposure stage of LI training rescued the LI of CFC in *Cacna1c*^+/−^ animals tested 24 h, 7 days and 21 days after CFC training (Fig. 5G, H). To assess whether intrahippocampal infusion had non-specific confounding effects on hippocampal function, animals received a second CFC in a separate novel context (context B) in the absence of any infusion, 22 or 23 days later. In the novel context B, animals expressed equivalent levels of freezing responses to context and normal hippocampal-dependent CFC and fear memory (Fig. 5G, H). LM22B-10 infused rats showed intact and context-specific LI of CFC when returned to the original conditioned context at LTM4 (Fig. 5G, H). Therefore, intrahippocampal infusion of LM22B-10 rescued LI without deleterious effects on hippocampal function. In a separate cohort of *Cacna1c*^+/−^ rats, intra-peritoneal injection of LM22B-10 throughout the LI procedure also rescued LI of CFC (Fig. S16).

Together, these findings show that activation of the ERK signalling pathway in the dorsal hippocampus during LI pre-exposure is sufficient to rescue the behavioural deficits seen in *Cacna1c*^+/−^ rats. This effect can be achieved with both intrahippocampal administration of LM22B-10 and with systemic dosing.

## Discussion

Advances in psychiatric genomics have consistently revealed association of schizophrenia, bipolar disorder and related neurodevelopmental disorders with genetic variation in VGCCs and in particular *CACNA1C*. Understanding the impact of genetic variation in the associated loci is critical for gaining mechanistic insight into these conditions and development of new therapies.

Psychotic symptoms pathognomonic of schizophrenia and frequent in bipolar disorder have been associated with altered learning about associations between environmental stimuli [18–20], leading to aberrant attribution of importance (or salience) to irrelevant events [36]. This aberrant learning can be objectivized by the LI procedure, which requires hippocampal and mesolimbic dopaminergic functional integrity [18, 37] and is affected in psychosis [17]. Consistent with impairments seen in psychotic patients, we found that low dosage of *Cacna1c* produced a marked deficit in contextual LI despite normal contextual fear conditioning. Our observations agree with previous studies of homozygous deletions of *Cacna1c* or *Cacna1d* (encoding the pore-forming subunits of Ca_V_1.2 and Ca_V_1.3 L-VGCCs, respectively) showing that Ca_V_1.3, but not Ca_V_1.2, is essential for acquisition of fear associations [35, 62]. Together with evidence from brain-specific knock-out studies [24, 63] our findings support a requirement for Ca_V_1.2, but not Ca_V_1.3, in context discrimination. The disruption of LI in wild-type animals and rescue in *Cacna1c*^*+/−*^ littermates by intrahippocampal drugs implicates the hippocampus as a major lesion site in our model. Therefore, hippocampal encoding of contextual information needed for LI may be particularly susceptible to alterations in *Cacna1c* dosage. Further studies are needed into potential upstream changes and the impact of this hippocampal dysfunction on mesolimbic dopamine signalling [16, 64] in our model.

Formation of novel context representations in the hippocampus involves synaptic LTP at excitatory afferents from EC and CA3 area during theta-burst firing [53]. We hypothesized that the low Ca_V_1.2 dosage in *Cacna1c*^*+/−*^ rats impacts on both LTP and the coordination of synaptic activity in CA1. Associative LTP is induced during theta-burst firing (TBP-LTP) by temporally coordinated pre- and postsynaptic neuronal activity and the activation of postsynaptic NMDARs and VGCCs [25, 26, 44]. In *Cacna1c*^+/−^ animals, TBP-LTP was impaired at SC-CA1 pathway although SC-CA1 synapses are capable of L-VGCC-independent LTP. This distinction mitigates against spine abnormalities as principal cause for the plasticity deficit.

To understand the causes of the SC-CA1 TBP-LTP deficit we investigated excitability and spine calcium signalling in *Cacna1c*^+/−^ CA1 pyramidal neurons. *Cacna1c*^+/−^ neurons had significantly reduced broadening of somatic APs during theta-burst-like firing, replicated in wild-type cells by L-VGCC block with isradipine, confirming a role for L-VGCCs. Consistent with weaker dendritic Ca^2+^ signals elicited by narrower APs [51] we observed inefficient summation of spine Ca^2+^ signals at SC-CA1 synapses during AP bursts in *Cacna1c*^+/−^ neurons. Our findings reveal a mechanism by which *Cacna1c* hemizygosity causes inadequate AP-triggered spine Ca^2+^ signalling disrupting the pre- and postsynaptic spiking association necessary for LTP [26]. A necessary pharmacological dissection of the relative contributions of Ca_V_1.2 and Ca_V_1.3 in these processes depends on the advent of isoform-selective antagonists, which are not yet available [33].

In *Cacna1c*^*+/−*^ animals TBP-LTP was preserved at the other major excitatory input, TA-CA1. This dissociation suggests that *Cacna1c* hemizygosity does not affect L-VGCC-dependent mechanisms in distal dendrites, where local spiking is more important for LTP [25]. Our results suggest an impaired processing of conjunctive EC and CA3 inputs to CA1 leading to altered novel context representations in *Cacna1c*^*+/−*^ animals.

Network synchronization in CA1 may contribute to the establishment of hippocampal memory traces by providing adequate temporal coordination between pre- and postsynaptic spiking [21]. PAC between CA1 theta and slow-gamma oscillations in *Cacna1c*^*+/−*^ rats was reduced specifically during exploration of a novel environment, indicating a mis-coupling between CA1 neurons and excitatory input from CA3 [55]. Theta–gamma PAC alterations across brain regions observed in animal models of psychosis [65] and schizophrenic patients [66], may reflect wider network deficits in psychoses.

One behavioural consequence of low *Cacna1c* dosage is a contextual LI deficit. Nevertheless, *Cacna1c*^*+/−*^ rats are apparently able to form contextual fear memories. This suggests that the ability to form context representations or associative contextual memory—an event or non-event in the context—is altered but not absent in our model. The successful retrieval and behavioural expression of such memories depends on reactivation of firing activity in distinct sparse hippocampal neuronal ensembles or engrams formed during learning [67, 68]. Altered engram formation may impair engram indexing function [68, 69], which may normally contribute to retrieval of the context-no event memory during conditioning, or retrieval of a specific memory during recall.

At molecular level, L-VGCC-mediated Ca^2+^ signalling imparts location and temporal specificity for excitation–transcription coupling and activation of ERK signalling [29, 30]. ERK is necessary for early [27, 28] and late, protein synthesis dependent, LTP and memory [70]. We found a markedly decreased activation of ERK and CREB in *Cacna1c*^+/−^ hippocampi, despite unchanged hippocampal cytoarchitecture [3].

Taken together our results show that *Cacna1c* hemizygosity impairs hippocampal function and ERK signalling-mediated excitation–transcription coupling likely to result in behavioural deficits observed in *Cacna1c*^*+/−*^ animals. Our findings implicate constitutively dysregulated *Cacna1c* expression in psychiatric risk, in line with altered cognitive functions and synaptic plasticity following embryonic ablation of *Cacna1c* in glutamatergic neurons [71]. An important future aim is to determine with greater confidence the contribution of specific cell types to these phenotypes.

Recent studies support the targeting of neurotrophin receptors using small-molecule BDNF mimetics as potential therapeutic strategy in neuropsychiatric and neurodegenerative disorders [72, 73]. We tested the effects of the small-molecule TrkB/TrkC co-activator LM22B-10 with BDNF mimetic activity in vivo [61]. In *Cacna1c*^*+/−*^ animals, direct application of LM22B-10 on hippocampal slices rescued synaptic plasticity, as predicted by ERK’s role in early-phase LTP [27, 28, 74]. Hippocampal infusion of LM22B-10 during context pre-exposure was sufficient to rescue LI, suggesting that context representations formed during pre-exposure are stable without requiring ongoing treatment. Systemic treatment with LM22B-10 rescued hippocampal molecular and plasticity changes and restored normal LI behaviour in *Cacna1c*^+/−^ rats. Future work will focus on effects of LM22B-10 on network activity in vivo, not addressed in this study, and translational biomarkers as provided by brain imaging.

In conclusion, by adopting a genomically informed approach to investigate pathological processes associated with genetic risk for neuropsychiatric disorders we show that *Cacna1c* hemizygosity impairs selective forms of associative learning, hippocampal synaptic plasticity, network synchronization, and ERK signalling-mediated excitation–transcription coupling. In addition, our work supports investigating the potential benefit of drugs targeting neurotrophin receptor signalling in psychiatric disorders.

## Supplementary information

Supplementary information

Supplementary Table S2

Supplementary Table S3
