# Supplementary material for: Neurotrophin receptor activation rescues cognitive and synaptic abnormalities caused by hemizygosity of the psychiatric risk gene Cacna1c
Source: Mol Psychiatry. 2021 Feb 17;26(6):1748–60. doi: 10.1038/s41380-020-01001-0 (PMC8440217; doi:10.1038/s41380-020-01001-0)
Supplement: Supplementary file 1 — Supplementary information [file 41380_2020_1001_MOESM1_ESM.pdf]

Supplementary Information

Neurotrophin receptor activation rescues cognitive and synaptic abnormalities caused by hemizygosity of the psychiatric risk gene *Cacna1c*.

Cezar M. Tigaret, Tzu-Ching E. Lin, Edward R. Morrell, Lucy Sykes, Anna L. Moon, Michael C. O'Donovan, Michael J. Owen, Lawrence S. Wilkinson, Matthew W. Jones, Kerrie L. Thomas, Jeremy Hall.

Supplementary Materials and Methods

Figure S1. LI of CFM deficit in *Cacna1c*$^{+/-}$ animals replicated in a variation of the LI paradigm.

Figure S2. Intrahippocampal infusion of L-type VGCC antagonist diltiazem disrupts components of contextual fear memory (Lister Hooded rats).

Figure S3. The deficit of TBP-induced SC-CA1 LTP is present in both male and female *Cacna1c*$^{+/-}$ rats.

Figure S4. NMDAR and L-VGCC dependence of LTP at SC-CA1 and TA-CA1 synapses in *Cacna1c*$^{+/+}$ *ex vivo* slices.

Figure S5. Paired-pulse facilitation shows no genotype effect on transmitter release at SC-CA1 and TA-CA1 synapses.

Figure S6. Isradipine-sensitive whole-cell Ca^{2+} currents in *Cacna1c*$^{+/-}$ vs *Cacna1c*$^{+/+}$ CA1 pyramidal neurons.

Figure S7. Reduced frequency-dependent broadening of somatic action potentials in CA1 pyramidal neurons from *Cacna1c*$^{+/-}$ animals.

Figure S8. Location of spines imaged for calcium transients triggered by somatic action potentials.

Figure S9. Attenuation of action potential-triggered spine calcium transients with distance from soma in CA1 pyramidal neurons.

Figure S10. Summation of action potential-triggered spine calcium transients with the number of action potentials.

Figure S11. Local field potential recordings in dorsal CA1 during novel vs familiar track behavioural protocol.

Figure S12. Behaviour on the familiar and novel linear track.

Figure S13. Reduced basal levels of phosphorylated ERK and phosphorylated CREB in the hippocampus of *Cacna1c*$^{+/-}$ rats.

Figure S14. *Cacna1c*$^{+/-}$ and *Cacna1c*$^{+/+}$ animals have similar hippocampal morphology and cellular densities in hippocampal subfields.

Figure S15. Rescue of basal levels of phosphorylated ERK and phosphorylated CREB in the dorsal hippocampus of *Cacna1c*$^{+/-}$ rats with systemic administration of LM22B-10 (related to Figure 5A).

Figure S16. Systemic administration of LM22B-10 rescues LI of CFC in *Cacna1c*$^{+/-}$ animals

Table S1. Passive membrane and action potential firing properties of CA1 pyramidal neurons in *Cacna1c*$^{+/+}$ and *Cacna1c*$^{+/-}$ littermates.

Table S2: Statistical comparisons of action potential-triggered spine calcium transients by regions of distance from soma.

Table S3: P-values for pairwise comparisons of action potential-triggered spine calcium transients in *Cacna1c*$^{+/+}$ *vs Cacna1c*$^{+/-}$ CA1 pyramidal neurons.

Supplementary Materials and methods.

Study design and sample sizes.

We used a *Cacna1c*$^{+/-}$ rat model in a multi-disciplinary, controlled laboratory experiment design with the objective to characterize the impact of reduced dosage of the psychiatric risk gene *CACNA1C* on behaviour, synaptic and circuit function and to explore ways to rescue the observed deficits. LI of CFC was chosen as the central behavioural paradigm of the study because it explores hippocampal-dependent associative learning implicated in cognitive functions translationally relevant to psychoses[1-5]. The *in vivo* and *ex vivo* electrophysiology and two-photon imaging experiments in the dorsal hippocampal CA1 subfield were informed by the observed context-sensitive behavioural deficits in *Cacna1c*$^{+/-}$ rats. These experiments were designed to investigate the impact of *Cacna1c* heterozygosity on hippocampal neural circuit synchronization, synaptic plasticity, excitability, and spine Ca^{2+} signalling as potential mechanisms underlying the behavioural deficit. The immunohistochemistry experiments were conducted to determine the impact of reduced *Cacna1c* dosage on the ERK/CREB signalling pathways downstream L-type VGCCs which has been implicated in synaptic plasticity, associative learning, and context-dependent learning[6-8]. For the rescue experiments we administered a small molecule TrkB/TrkC receptor agonist previously characterized for ERK pathway activation activity and neurotrophic effects after systemic administration[9] either intrahippocampally or systemically (intra-peritoneal injections). When given peripherally, the agonist (or vehicle) was administered prior to each behavioural manipulation to account for non-specific state dependent effects[10].

Sample sizes were determined by power analysis using G*Power software, with $\alpha = 0.05$ and $\beta = 0.2$ based on effect sizes (Hodge's g) estimated from published data including from our lab. Previous behavioural analyses in our lab using similar experimental design and genotypes[11] suggested a minimum n=6 animals per group, for a mean Hedge's g of 1.2. For

LTP experiments, a mean Hodge's g of 1.6 for the difference in Test vs Control pathway[12] yielded a minimum n = 6 experimental units (cells) per group. To detect significant differences in AP durations between the two genotypes we used the effect of the BK channel antagonist iberiotoxin as a proxy for changes in AP duration[13,14], with an estimated Hodge's g of 0.9 yielding a minimum n = 21 neurons per group. A minimum n = 12 spines needed to detect significant differences in Ca^{2+} transients was taken directly from our previous research[12,15].

Behavioural data was analysed using repeated measures ANOVA. The *ex vivo* electrophysiology and two-photon imaging data were analysed using two-way ordinal regression with repeated measures and a generalized linear model to minimize Type I errors due to nested data. Details of statistical analyses are given under each corresponding section below.

Behavioural paradigms.

<u>Contextual fear conditioning (CFC), contextual fear memory (CFM).</u> A paradigm of a single footshock exposure in a novel context was used[16]. During 3-min CFC training trial, each animal received a single scramble footshock (US: 0.5mA, 2s) after being placed in a conditioned chamber (context) for 2 min. Animals were returned to home cage after conditioning. CFM retrieval was tested by measuring the animal's freezing response upon returning to the conditioned chamber for 2 min. Retrieval tests were performed 3 h (short-term memory, STM), 24 h (long-term memory LTM1), 7 days (LTM2) and 21 days (LMT3) and 23 days (LTM4) after CFC training. Intrahippocampal LM22B-10 infusion rats received additional CFC training and CFM retrieval in an alternative context (context B) on Days 22 and 23 after the first CFC training. The freezing behaviour was used as measure of conditioned fear. One unit of freezing response was defined as the continuous absence of movement other than respiratory motion in 1 s sampled every 10 s. Freezing responses were

video-recorded during each trial and quantified as percentage of time spent freezing by an observer blind to the experimental group.

Latent inhibition of contextual fear conditioning (LI of CFC). LI training consisted of animal pre-exposure (4 h, PE) to the conditioned context, either 24h or 48 h prior to CFC training.

Apparatus for behavioural testing. All sessions were undertaken in standard modular test chambers for rats (interior dimensions: 30.5 cm x 24.1 cm x 21.0 cm) placed inside sound attenuating chambers 55.9 cm x 55.9 cm x 35.6 cm (Med Associates Inc., Vermont, USA). Two test chambers were used as a standard context for single CFC, one consists of standard transparent Perspex walls, and the other chamber was decorated with black and white stars wallpaper on the Perspex. Both chambers were equipped with 19 equally spaced metal bars placed 1.6 cm above the floor, for the delivery of a 0.5 mA current (footshock) under control by a stand-alone aversive stimulator/scrambler (Med Associates Inc., Vermont, USA). Rats were placed in either of the chamber for single CFC training. The chambers were wiped with 50% EtOH prior placing the animals in the chamber. For rats that received additional CFC training in an alternative context, had they received original CFC in transparent Perspex chamber (context A) would be placed in star decorated chamber, wiped with 5% pepper mint water (context B) for the second CFC training. The programmes for each session were controlled through a Med-PC version IV research control and data acquisition system (Med Associates Inc., Vermont, USA). Behaviour was video recorded with cameras (JSP Electronics Ltd, China) positioned centrally above the chambers, digitized, and analysed offline using Numeroscope software (Viewpoint, France). For each training or retrieval session, animals were individually transferred between from home cages and the testing room in the same large transport box.

Statistical analysis of behavioural data. Data were analysed with repeated measures analysis of variance (ANOVA) and Mauchly’s test for sphericity, with Greenhouse–Geisser correction

applied when necessary. ANOVA for drug experiments included only post-treatment data. Pairwise comparisons with Bonferroni correction were performed when factors had significant interaction.

In vivo electrophysiology.

Surgical Procedure. All surgery was carried out aseptically to minimize the risk of infection. Animals were anaesthetized with isoflurane in oxygen (maintained at 1.5-2%) and head-fixed in a stereotaxic frame (Kopf model 1900). After exposing the skull surface, a 2.5mm craniotomy was made using a tungsten carbide burr (3.6mm posterior to bregma and 2.5mm lateral to the midline) and a 20-tetrode (16 recording electrodes and 4 reference electrodes) microdrive was lowered into the craniotomy (Fig. S11A). The microdrive was fixed in place using dental cement (dePuY). Animals were given buprenorphine (0.025mg/kg) following surgery, and weight and water consumption were monitored for at least one week afterwards. Over a duration of 2-4 weeks tetrodes were gradually lowered (~20-40µM a day) to the pyramidal cell layer of CA1 (verified by the presence of sharp-wave ripples and bursting spike activity).

Data Acquisition. Recordings were made using a Digital Lynx SX system (Neuralynx). Local field potentials were sampled at 1kHz and filtered between 0.1 and 475Hz). Animal position was monitored with an overhead camera and an LED attached to the headstage.

Training. Animals were trained to run back and forth on a 175cm linear track over a period of 1-4 weeks. During this period animals were food restricted to 85% of their initial weight. Behavioural experiments were carried out once tetrodes were in place in the pyramidal cell layer of dorsal CA1 and animals had reached a minimum criterion of 10 complete circuits on the linear track.

Familiar Track Behavioural Protocol (Fig. S11B). Animals were placed in a sleep-box (a sound-attenuating chamber) and left to rest for approximately 30 minutes prior to track running. After the pre-sleep period animals were allowed to run freely for sucrose rewards. Following the run, animals were placed back in the sleep-box for a further 30 minutes. Animal position and electrophysiological signals were continuously recorded throughout the behavioural session.

Novel Track Behavioural Protocol (Fig. S11C). Following at least one recording on the familiar track and at least a day after the familiar track session, animals were subject to the novel position behavioural protocol. The pre and post sleep protocol here did not differ to that of the familiar track behavioural sessions. For the track sessions animals were placed on the familiar track for approximately 10 minutes to take baseline recordings. The animals were then taken off the track, the track was rotated 45° and animals were immediately placed back on and recorded while they now freely ran through a different portion of the recording room.

Data Analysis. All data analysis of in vivo electrophysiology was carried out in MatLab (Mathworks). Local field potential signals were taken from periods of track running over 35cm/s and all analysis was performed on these segments of local field potential. To control for individual animal differences in the number of circuits on the familiar track compared to the novel track, a matched number of runs were pseudo-randomly selected from the session containing more runs.

Power Spectral Analysis. All power spectral estimates were calculated using multi-taper spectral analysis (Chronux toolbox) with a window length of 2.5s, a time-bandwidth product of 3 and 5 tapers.

Phase-Amplitude Coupling. Phase-amplitude coupling was detected using a PAC toolbox applying the modulation index measure[17]. In brief this method combines the amplitude

envelope of the local field potential filtered at a high frequency band with the phase values of the signal filtered at a lower band to form a composite signal. The mean of this signal provides a measure of the strength of coupling between the amplitude of the higher frequency band with the phase of the lower frequency band.

Statistical analysis of *in vivo* electrophysiology data. All group comparisons were carried out using student's t-test.

Ex vivo electrophysiology.

Slice preparation and recordings. Acute transverse hippocampal slices were obtained from 3 – 6 months old male and female SD-Cacna1c rats after being given a lethal dose of isoflurane inhalation. The hippocampi were dissected in ice-cold slicing solution (in mM: 110 Choline chloride, 25 glucose, 25 $NaHCO_3$, 2.5 KCl, 1.25 NaH_2PO_4, 0.5 $CaCl_2$, 7 $MgCl_2$, 11.6 L-Ascorbic acid, 3 pyruvic acid), then mounted on agar blocks and cut in 400 µm thick slices on a Microm HM 650V vibratome (Thermo-Scientific). Slices were incubated in recovery solution (in mM: 93 N-methyl-D-glucamine, 25 glucose, 30 $NaHCO_3$, 20 HEPES, 2.5 KCl, 1.2 NaH_2PO_4, 0.5 $CaCl_2$, 7 $MgCl_2$, 5 L-Ascorbic acid, 3 pyruvic acid, 12 N-acetyl-cysteine, 2 thiourea) at 35 °C for 5 min, then incubated in artificial CSF (aCSF, in mM: 119 NaCl, 10 glucose, 26.2 $NaHCO_3$, 2.5 KCl, 1 NaH_2PO_4, 2.5 $CaCl_2$ and 1.3 MCl_2), at 35 °C for 30 min. Slices were then stored in aCSF at room temperature until use. Solutions were equilibrated with 95% O_2 and 5% CO_2 and had osmolarity of 300 – 310 mOsm. Whole-cell patch-clamp recordings were made from CA1 pyramidal neurons visualized under differential interference contrast (Olympus BX-51 WI) in a submerged recording chamber superfused with aCSF (~ 2 ml/min) at 35 °C. Patch electrodes (4 – 6 MΩ) were pulled from borosilicate filamented capillaries on a Model P-1000 Flaming/Brown micropipette pulse (Sutter Instruments) and filled with intracellular solution (in mM: 117 $KMeSO_3$, 8 NaCl, 1 $MgCl_2$, 10 HEPES, 4 MgATP, 0.3 NaGTP, pH 7.2, 280 mOsm, 0.2 EGTA). For the experiments in Fig 2f-k

involving tonic postsynaptic depolarization the firing of action potentials was blocked by substituting $CsMeSO_3$ for $KMeSO_3$ and adding 5 mM QX-314 to the internal solution (pH was adjusted with CsOH). Recordings were made with a Multiclamp 700B amplifier and digitized with an Axon Digidata 1550 Data Acquisition System and pClamp 10.7 software (Molecular Devices).

<u>Synaptic plasticity experiments.</u> Synaptic currents were recorded in voltage-clamp (-70 mV), and the aCSF was supplemented with 50 µM picrotoxin to block $GABA_A$ receptors. Synaptic responses were evoked with 0.1-1 ms square pulses delivered through bipolar tungsten electrodes (100 kΩ, MicroProbes) placed on opposite sides of the recorded cell, in *stratum radiatum* (schematic in Fig 2A,E) or in *stratum lacunosum-moleculare* (schematic in Fig 2I). For synaptic plasticity experiments synaptic currents elicited at 0.1 Hz alternatively in Test and Control pathways were filtered at 4 kHz and digitized at 10 kHz. When used, back-propagated postsynaptic action potentials (bAPs) were evoked by somatic current injection (2 nA, 2 ms). Membrane voltage was not corrected for liquid junction potential, calculated at -9 mV. The pathways were tuned in current clamp to evoke sub-threshold EPSPs prior to baseline recording. Pathway independence and paired-pulse facilitation were tested using paired stimuli delivered at 50 ms intervals (paired-pulse protocols). Paired-pulse facilitations was then calculated as the amplitude ratio between the second and the first synaptic response. Data was analysed offline using routines written in Python. Consecutive excitatory postsynaptic currents (EPSC) in each pathway were averaged every minute and their amplitudes normalised to the average of 5 min before the application of a conditioning protocol to the Test pathway (baseline), while the Control pathway was left unperturbed. The mean EPSC amplitude in the Test and Control pathway at 30 – 35 min after conditioning was normalized to the mean EPSC amplitude during the 5 min baseline. The theta-burst pairing (TBP, Fig. 2A-D and I-L, S3, S4) conditioning protocol consisted of three 1s trains of short

pre- and postsynaptic bursts delivered at 5 Hz every 10s intervals. Each burst consisted of five of coincident presynaptic stimuli and postsynaptic bAPs at 100 Hz. The low-frequency stimulation pairing (LFS-Pairing, Fig. 2E-H) protocol consisted of a train of presynaptic stimulations delivered at 2 Hz for 90 s, paired with tonic postsynaptic depolarization at 0 mV. Series resistance was monitored throughout the recording and cells with series resistance > 30 MΩ or showing > 20% change in series resistance were discarded. When used, drugs were continuously in the perfusate throughout the experiment.

Voltage-clamp experiments for whole-cell Ca^{2+} currents.

Recordings were performed in the aCSF (see Supplementary Methods) containing the K^+ channel blockers TEA (124 mM) and 4-aminopyridine (4-AP, 15 mM), and 1µM tetrodotoxin, in the absence (Control) or presence of L-VGCC antagonist isradipine (Isr, 10µM). The pipette solution (pH 7.2, 280 mOsm) contained (in mM): Cs gluconate (120), TEA-Cl (20), EGTA (1), HEPES (10), MgATP (4) and NaGTP (0.3). Series resistance and capacitance were compensated after establishing whole-cell configuration and checked periodically. To minimize clamping artifacts inherent in recordings from mature hippocampal neurons in slices, whole-cell Ca^{2+} currents (I_{Ca}) were evoked with voltage ramps[18] (schematic in panel A). Cells were held at -70 mV, then inward I_{Ca} currents were evoked by steadily increasing the membrane voltage from -80 mV to +80 mV over 160 ms. Leak currents were subtracted during acquisition using the P/6 method (Clampex sofware).

The current-voltage relationships were fitted off-line with the product of a squared Boltzmann function with Goldman-Hodgkin-Katz current equation[19]:

$$I(V) = \frac{1}{\left[1 + e^{-\alpha(V-V_0)F/RT}\right]^2} \times \frac{\beta V\left\{[Ca]_i - \left([Ca]_o e^{-2VF/RT}\right)\right\}}{1 - e^{-2VF/RT}}$$

where $F/_{RT} = 0.0379\ mV^{-1}$ at 33°C, V_o is the shift of the Boltzmann function (left factor in the equation), α and β are, respectively, slope and scale factors, and $[Ca]_o$ and $[Ca]_i$ are, respectively, extracellular and intracellular Ca^{2+} concentrations. The fitted parameters (α, β and $[Ca]_i$ and V_o) were used to compute the I_{Ca} integral (area under the I_{Ca} curve) on the interval between -40 mV and +25 mV.

I_{Ca} recorded in the presence of Isr were normalized off-line to the peak inward I_{Ca} in the absence of the drug (Control), and the Isr-sensitive fraction of peak I_{Ca} was calculated as 1 minus the maximal peak-normalized I_{Ca} during Isr. Normalized currents were fitted as described above and the Isr-sensitive fraction of I_{Ca} integral was calculated as 1 minus the ratio of the peak-normalized I_{Ca} integrals during Isr and control.

Passive and active electrophysiological membrane properties. Membrane properties were determined from membrane potential (V_m) recordings during 500 ms square-wave current injections in whole-cell current clamp, using the amplifier bridge circuit[20]. The recordings were filtered at 6 kHz and digitized at 50 kHz. After determining the resting membrane potential (RMP), the membrane potential was set to -70 mV by a small inward current injection. The membrane time constant (τ_m), the hyperpolarization-activated "sag" potential (V_{sag}) and the input resistance (R_{in}) were determined current injections of -0.1 nA (schematic in Fig S6A). V_{sag} was measured as the difference between the V_m minimum and the steady-state hyperpolarization elicited by the injected current, and expressed as a percentage of the steady-state membrane voltage (V_{ss}); τ_m was determined from an exponential fit of the V_m curve between 10% – 95% from baseline to the V_{sag} minimum; R_{in} was calculated according to the Ohm's law (V=I×R) using the difference between the steady-state hyperpolarization (V_{ss}) and the resting membrane potential (RMP). Active membrane properties were determined from V_m recordings during a series of depolarizing current injections steps from 0 to +0.7 nA in 50 or 100 pA increments every 10 s, to elicit action potential (AP) firing (Fig.

S6B). The rheobase current (I_{rh}) was determined using exponential fits through strength-latency curves representing the injected current intensity as a function of spike latency[21]. AP waveforms were detected by thresholding the first-order derivative of the recordings, and V_m onset was determined as the membrane voltage where the rate of change of membrane potential (dV/dt) exceeded 20 V/s[22]. AP waveform duration was measured at one third from onset to peak, and the maximum rate of change (dV/dt) of the V_m during an AP was determined from the first-order derivative of the AP waveform. The instantaneous AP was calculated as the inverse of the inter-spike interval (ISI). To compare the relationship between AP durations and the firing rate, the AP trains recorded in each cell were grouped according to the instantaneous frequency at the 1st ISI, in four frequency bands (Fig. S6F-I) and the durations of individual APs were averaged across the trains in the same frequency band. Some cells did not fire AP trains with frequencies falling in all four bands. The number of cells which fired AP trains in the given frequency band are indicated in the legend of Fig. S6. When used, isradipine was added to the perfusate following an initial series of current injections ("Vehicle"), and recordings were restarted after 7-10 min of drug wash-in.

Statistical analysis of *ex vivo* electrophysiology data. In synaptic plasticity experiments, the normalized mean EPSC amplitudes at 30-35 min after conditioning were compared with Test/Control pathway (within-subjects factor) and genotype or drug treatment (between-subjects factor). The effect of genotype or drugs on LTP induction were tested for significance using two-way ordinal regression and analysis of deviance (ANODE). Passive membrane properties were compared using two-sample Mann-Whitney U test between genotypes against the null hypothesis of no difference between genotype-specific means. AP parameters for the first five or 10 APs during spike train discharges were compared using two-way ordinal regression with repeated measures for AP number and factors of genotype or first ISI frequency calculated as the reciprocal of first inter-spike interval.

Two-photon spine Ca^{2+} imaging.

Data acquisition. Spine Ca^{2+} imaging was performed on a galvanometer-based two-photon laser scanning system (Ultima In Vitro Multiphoton Microscope System with Prairie View v.5.0 software, Bruker France – Nano Surfaces) in CA1 pyramidal neurons in *ex vivo* transverse hippocampal slices. Patch electrodes were filled with intracellular solution (in mM: 117 $KMeSO_3$, 8 NaCl, 1 $MgCl_2$, 10 HEPES, 4 MgATP, 0.3 NaGTP, pH 7.2, 280 mOsm) supplemented with the medium affinity fluorescent Ca^{2+} indicator (Fluo-5F 200 µM; Life Technologies) and a reference fluorescent dye (AlexaFluor 594, 30 µM; Life Technologies). EGTA was omitted to avoid introducing additional Ca^{2+} buffering capacity in the cells. Fluorescence was excited with a Chameleon Ultra II Ti:Sapphire laser (Coherent) tuned at 810 nm. Ca^{2+} transients elicited by back-propagated action potentials (APCaTs) were imaged in dendritic spines in the *stratum radiatum* using line scanning mode[23] with a 60× water-immersion objective. After establishing whole-cell configuration in voltage-clamp, the cells were switched to current-clamp, the RMP was measured, and the membrane voltage was set to -70 mV by inward current injection (-50 pA – 100 pA). Cells were dye-loaded for 10-15 min then APCaTs were recorded in line-scanning mode, in series of 1000 lines per second, for 1s. To minimize photodamage, line scans were acquired in batches of up to 20 repeats every 20 s. APCaTs were evoked by delivering either one, two, three or five APs by brief (2 nA, 2 ms) somatic current injections (Fig. 3). Between batches, the RMP was monitored and cells where the RMP exceeded -60 mV or varied by more than 10 mV were discarded. Cells were discarded if they showed signs of photodamage (localized swellings, sustained increase in resting Ca^{2+} fluorescence, or tonic increase in Ca^{2+} fluorescence after stimulus, without return to baseline levels). Within the batches, the line scan series were interleaved with respect to the number of delivered APs, to compensate for small variations in dye loading between the beginning and the end of the imaging session. The trajectory of the line scan was

set to image 3 – 10 spines per dendritic segment, on branches up to the third order in *stratum radiatum*. To minimize the risk of photodamage and surplus Ca^{2+} buffer capacity due to continuous dye accumulation we recorded spine APCaTs from up to three different dendritic locations and a single dendritic segment was imaged in each location. The total imaging time was limited to 50 – 60 min since break in. The locations of spines were selected pseudo-randomly with the constraint that the parent dendritic segments belong to distinct branches originating from the main trunk at least 30 µm apart (Fig. S7).

Data analysis. Fluorescence images were analysed off-line using software written in Python. Line scan images were de-noised and data from up to five replicates in each protocol were averaged for each spine, including failures. The small number of replicates in each protocol precluded reliable calculation of success rates. APCaT traces were then generated as the relative change in Fluo-5F fluorescence channel *versus* Alexa Fluor channel (ΔF/A) and fitted with an exponential rise and decay[23]. The APCaT peak ΔF/A amplitude and time integral values were derived from the fitted curves. The linearity of APCaT summation with increasing number of somatic APs was determined in each spine by normalizing the peak amplitude and time integral to the values of APCaTs elicited by single APs (1bAP). The distances from the soma to the parent dendritic segment of the imaged spines were determined *post hoc* using Simple Neurite Tracer[24] from three-dimensional reconstructions (Z-stacks) recorded in the AlexaFluor 594 channel at the end of the experiment. The spines located on the same segment were attributed the same distance from soma as the parent dendritic segment. The data were classified according to the somatic distance of their parent dendritic segment and grouped in three distance zones (in µm): 0 – 150, 150 – 250, and 250 – 400, with the majority of Schaffer collateral synapses being located in the nearest ~ 250 µm zone corresponding to the *Stratum radiatum*[25]. The distribution of spine locations is shown in Fig. S7.

Statistical analysis of two-photon Ca^{2+} imaging data. AP-elicited spine Ca^{2+} transients were compared within somatic distance zones using a generalized linear model with factors of genotype and number of APs, followed by analysis of variance (ANOVA). Tukey-adjusted p-values for post-hoc pairwise comparisons are indicated in figure legends together with the likelihood ratio chi square test values (LR), F statistic, and degrees of freedom for main effects and interactions.

Immunohistochemistry and DAPI staining.

Rats were given a lethal dose of Euthatal, i.p. and transcardially perfused with iced-cold saline and 4% PFA in phosphate buffer (PB). Brains were removed and post-fixed in the same fixative overnight and were then immersed in 30% sucrose in PB for 48 hours. Free floating coronal sections were cut at 35μm thickness on a freezing microtome and stored in cryoprotective solution in -20°C freezer prior to immunohistochemistry. For total and phospho-ERK (active ERK) immunostaining, anti-p44/42 MAPK (#4695) and anti-phospho-p44/42 MAPK (#4370) primary antibody (1:1000 for both antibodies, Cell Signalling) were used, respectively. The detection of the bound antibodies was carried out using a standard peroxidase-based method (ABC-kit, Vectastain), followed by DAB (Sigma-Aldrich) and 3% H_2O_2 solution. Sections were then mounted on poly-L lysine coated slides, dehydrated, and mounted in DPX mounting medium (Sigma-Aldrich). For CREB and phosphorylated CREB immunofluorescence staining, anti-CREB and anti-phospho-CREB primary antibody (CREB (#9197): 1:1000; pCREB (#9198): 1:500; Cell Signalling) were used. Alexa Fluor 488 anti-rabbit secondary antibody (1:1000, Invitrogen, UK) was used to label the cells. Nuclei were stained with DAPI (1:1000, Sigma-Aldrich). Sections were then mounted on poly-L lysine coated slides, dehydrated, and mounted with Mowiol mounting medium (Sigma-Aldrich). Images of the regions of interests were captured from both hemisphere from 3 to 4 sections (each 420μm apart) per animal. Coordinates of sections being processed were restricted to the

dorsal hippocampus between bregma -2.76 mm and -3.96 mm[26]. Immunofluorescence images were captured using a Leica DM6000B upright microscope equipped with an Olympus DP70 camera. Data (i.e., positive cells counts per μm^2) was quantified using Image/J-Fiji.

Statistical analysis of immunohistochemistry data. Analysis was performed using ANOVA followed by pairwise comparisons with Bonferroni correction if significant interactions between factors was found. Two statistical outliers (> ± 2 SD) were removed from the analysis of phosphorylated CREB and phosphorylated ERK (one *Cacna1c*$^{+/-}$ rat each, Fig. S13).

Intra-hippocampal infusion.

Rats were surgically implanted with stainless steel double guide cannula (Plastics One, UK, 22 gauge, 3 mm below pedestal) under general anaesthesia with Isoflurane (Isoflurane: 5% - 2%, flow rate: O_2: 0.8 litter/min), as described previously[27]. All surgery was carried out aseptically to minimize the risk of infection. Briefly, the double guide cannula was placed 3.8 mm apart and was aimed at the dorsal hippocampus 3.5mm posterior from the bregma. All rats were left to recover for a minimum of 6 days before behavioural experiments. Bilateral infusions via the chronically indwelling cannula were carried out in awake rats using a syringe pump, connected to injectors (28-gauge, projecting 1mm beyond the guide cannula) by polyethylene tubing. Diltiazem Hydrochloride (10 nmol/µl, DTZ, Sigma-Aldrich, UK) was dissolved in vehicle solution: 0.1M sterile phosphate-buffered saline (PBS), pH 7.2, treated with diethyl pyrocarbonate (DEPC). LM22B-10 (2µM, Tocris Bioscience, UK) was dissolved in vehicle PBS containing 0.1% DMSO. Each hemisphere received 1µl of drug solution or vehicle at the rate of 0.5µl per minute before the start of specific trials as indicated. The volume of LM22B-10 and PBS was 1µl per hemisphere and delivered at the rate of 0.5µl per minute. The injection cannula remained in place for further 2 mins to allow time for the diffusion of solutions away from the injection site before they were slowly

removed. Histological determination of the cannula placement using β-thionin staining of Nissl substance showed the placement of cannula in the dorsal hippocampus with minimal tissue damage.

Systemic administration of drugs.

LM22B-10 (Tocris Bioscience, UK) for peripheral injections was prepared as described[9]. The animals were weighted and the amount of drug necessary to deliver 25 mg/kg in 1 ml injection volume was dissolved in HCl then mixed with 5% Cremophor (Sigma-Aldrich) and 2X sterile PBS. The solution was adjusted for pH 5 then diluted with double deionized water to 1 X PBS and 2.5% Cremophor. The drug solution was injected via intra-peritoneally 60 mins prior to each training stage of LI or before immunohistochemical and *ex vivo* slices preparations. The dose of 25 mg/kg LM22B-10 in rats was derived[28] from the 50 mg/kg dose in mice at which a single i.p. bolus yields micromolar drug concentrations in brain tissue[9].

Code availability.

The software used for the analysis of two-photon imaging and electrophysiology data is available upon request, from Prof. Jeremy Hall (Cardiff University) and Prof. Matthew W. Jones (University of Bristol).

Supplementary References:

1.Lubow RE, Weiner I, Schlossberg A, Baruch I. *Bulletin of the Psychonomic Society* 1987; **25**(6)**:** 464-7.
2.Gray JA. *Schizophr Bull* 1998; **24**(2)**:** 249-66.
3.Hemsley DR. *Neurosci Biobehav Rev* 2005; **29**(6)**:** 977-88.
4.Maren S, Phan KL, Liberzon I. *Nat Rev Neurosci* 2013; **14**(6)**:** 417-28.
5.Gill KM, Miller SA, Grace AA. *Schizophrenia Research* 2018; **195:** 343-52.
6.Wheeler DG, Barrett CF, Groth RD, Safa P, Tsien RW. *The Journal of Cell Biology* 2008; **183**(5)**:** 849-63.
7.Winder DG, Martin KC, Muzzio IA, Rohrer D, Chruscinski A, Kobilka B *et al. Neuron* 1999; **24**(3)**:** 715-26.
8.Chwang WB, O'Riordan KJ, Levenson JM, Sweatt JD. *Learning & Memory (Cold Spring Harbor, NY)* 2006; **13**(3)**:** 322-8.

9.Yang T, Massa SM, Tran KC, Simmons DA, Rajadas J, Zeng AY *et al.* *Neuropharmacology* 2016; **110**(Pt A)**:** 343-61.
10.Overton DA. In: Iversen LL, Iversen SD, Snyder SH (eds). *Drugs, Neurotransmitters, and Behavior*. Springer US: Boston, MA, 1984, pp 59-127.
11.Moon AL, Brydges NM, Wilkinson LS, Hall J, Thomas KL. *Schizophr Bull* 2020.
12.Tigaret CM, Olivo V, Sadowski J, Ashby MC, Mellor JR. *Nat Commun* 2016; **7:** 10289.
13.Bock T, Stuart GJ. *Frontiers in Cellular Neuroscience* 2016; **10:** 206.
14.Shao LR, Halvorsrud R, Borg-Graham L, Storm JF. *The Journal of Physiology* 1999; **521 Pt 1:** 135-46.
15.Tigaret CM, Chamberlain SEL, Sadowski J, Hall J, Ashby MC, Mellor JR. *J Neurosci* 2018; **38**(43)**:** 9252-62.
16.Fanselow MS. *Animal Learning & Behavior* 1990; **18**(3)**:** 264-70.
17.Onslow AC, Bogacz R, Jones MW. *Prog Biophys Mol Biol* 2011; **105**(1-2)**:** 49-57.
18.Moosmang S, Haider N, Klugbauer N, Adelsberger H, Langwieser N, Müller J *et al. The Journal of Neuroscience: The Official Journal of the Society for Neuroscience* 2005; **25**(43)**:** 9883-92.
19.Talbot MJ, Sayer RJ. *J Neurophysiol* 1996; **76**(3)**:** 2120-4.
20.Tamagnini F, Novelia J, Kerrigan TL, Brown JT, Tsaneva-Atanasova K, Randall AD. *Frontiers in Cellular Neuroscience* 2015; **9:** 372.
21.Frank K, Fuortes MG. *J Physiol* 1956; **134**(2)**:** 451-70.
22.Naundorf B, Wolf F, Volgushev M. *Nature* 2006; **440**(7087)**:** 1060-3.
23.Tigaret CM, Tsaneva-Atanasova K, Collingridge GL, Mellor JR. *Biophysical Journal* 2013; **104**(5)**:** 1006-17.
24.Longair MH, Baker DA, Armstrong JD. *Bioinformatics (Oxford, England)* 2011; **27**(17)**:** 2453-4.
25.Megías M, Emri Z, Freund TF, Gulyás AI. *Neuroscience* 2001; **102**(3)**:** 527-40.
26.Paxinos G, Watson C. *Paxino's and Watson's The rat brain in stereotaxic coordinates*. Seventh edition. edn. Elsevier/AP, Academic Press is an imprint of Elsevier: Amsterdam ; Boston, 2014, 1 volume (unpaged)pp.
27.Lee JL, Everitt BJ, Thomas KL. *Science* 2004; **304**(5672)**:** 839-43.
28.Nair A, Morsy MA, Jacob S. *Drug Dev Res* 2018; **79**(8)**:** 373-82.

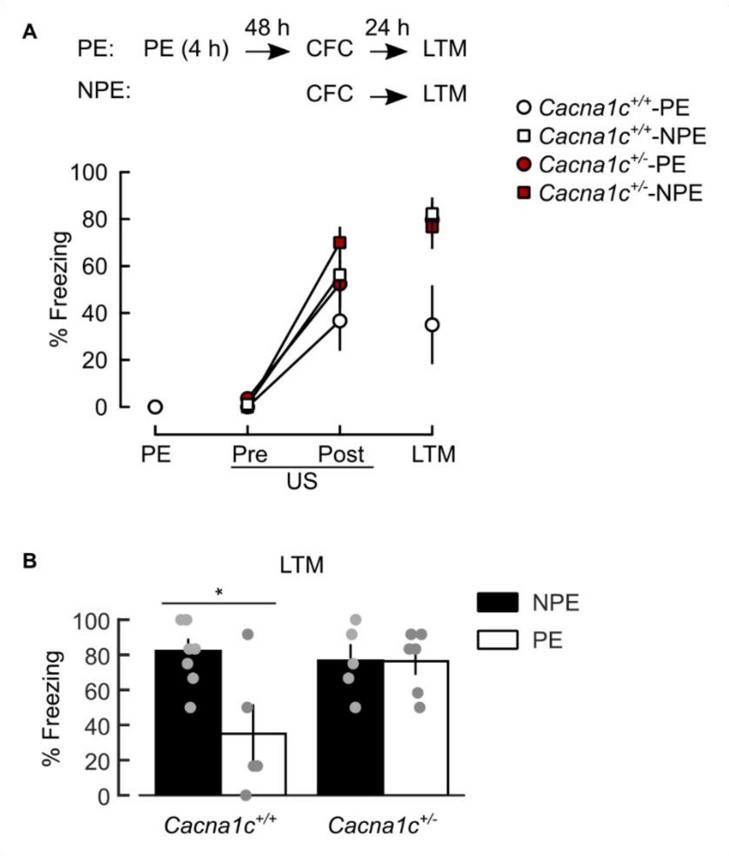

Figure S1. LI of CFM deficit in *Cacna1c*$^{+/-}$ animals replicated in a separate cohort of animals using a variation of the LI paradigm. (**A**) *Top:* schematics of the behavioural protocols; PE: 4h context pre-exposure (*Cacna1c*$^{+/+}$-PE: n=5; *Cacna1c*$^{+/-}$-PE: n=7); CFC: contextual fear conditioning comprising Pre-US and Post-US epochs before and after footshock (US), respectively; LTM: long-term memory test. NPE: non-pre-exposed (*Cacna1c*$^{+/+}$-NPE: n=8; *Cacna1c*$^{+/-}$-NPE: n=5). *Bottom*: Freezing responses at each session. The results showed a significant effect of trial ($F_{(2,42)}$=74.671, p<0.0001), genotype (*Cacna1c*$^{+/+}$ *versus Cacna1c*$^{+/-}$: $F_{(1,21)}$=6.521, p=0.018) and pre-exposure (PE *versus* NPE: $F_{(1,21)}$=8.017, p=0.01), and a marginal interaction between group × pre-exposure interaction ($F_{(1,21)}$=4.182, p=0.054). (**B**) Summary of freezing responses at LTM. There was significant effect of pre-exposure ($F_{(1,21)}$=5.453, p=.003) and genotype × pre-exposure interaction ($F_{(1,21)}$=7.088, p=.015) at LTM. In the *Cacna1c*$^{+/+}$ rats, context pre-exposure reduced the conditioned freezing response at LTM, indicating LI of CFC. The high levels of freezing response in pre-exposed *Cacna1c*$^{+/-}$ rats were similar to those seen at LTM1 in the paradigm using 24 h delay between PE and CFC (Figure 1B). Data shown as means ± SEM. *p <0.05 (two-way repeated measures ANOVA followed by Tukey adjustment of p-values for contrasts).

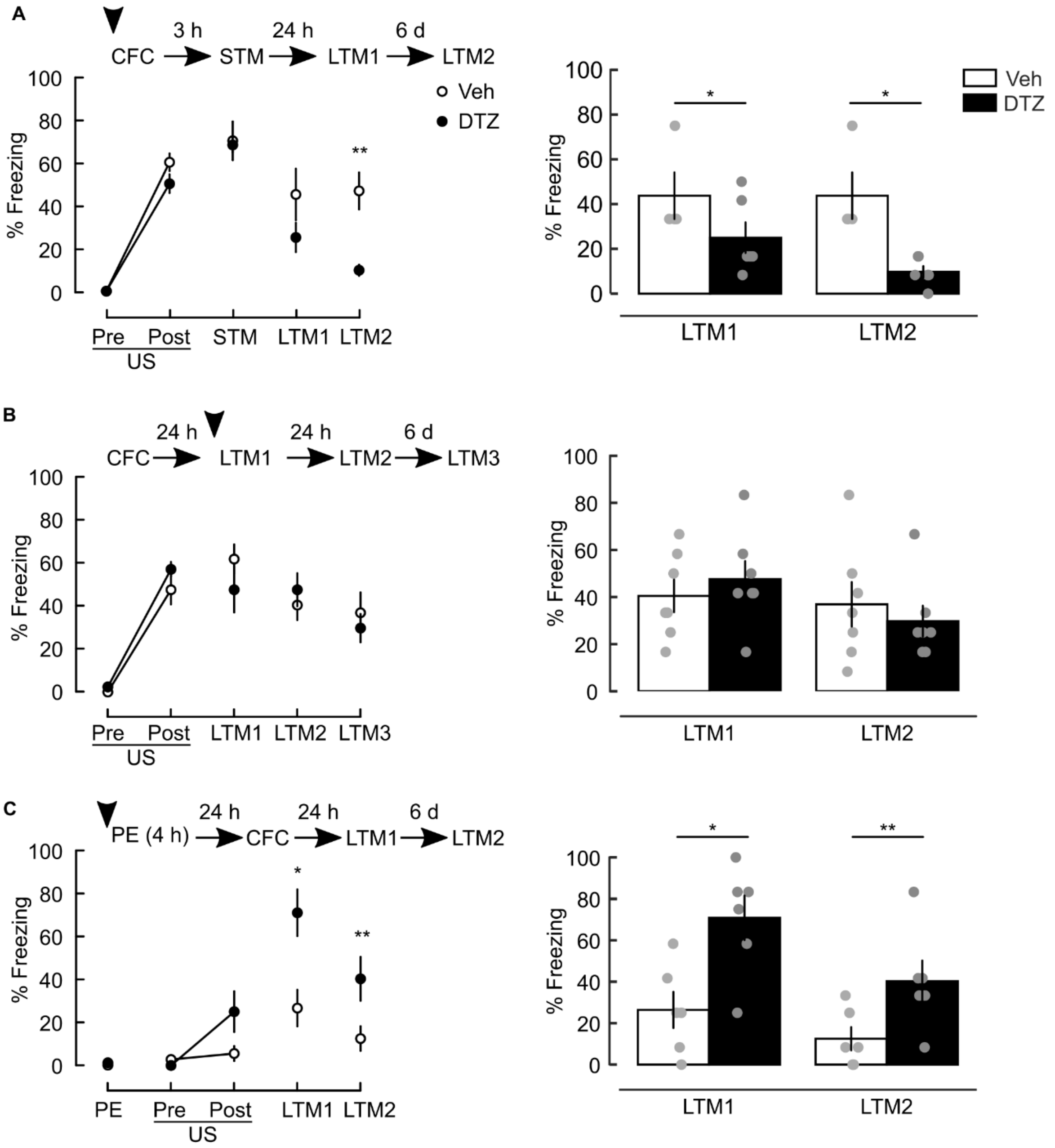

Figure S2. Intrahippocampal infusion of the L-type VGCC antagonist diltiazem disrupts components of contextual fear memory (Lister Hooded rats). All panels: *Left*: Plots of freezing response during the trials of the CFC paradigms, with session schematics at top. PE: 4h context pre-exposure; CFC: contextual fear conditioning comprising Pre-Us and Post-US epochs before and after footshock (US), respectively; STM, LTM1, LTM2, and LTM3: short-term and long-term contextual fear memory (CFM) tests. Arrowheads: bilateral infusion of 1µl diltiazem (DTZ, 10 nmol/µl) or vehicle (Veh) in dorsal hippocampus, 40 min before the indicated session. *Right*: Summary of the freezing responses at LTM tests. (**A**) DTZ infusion before CFC blocks the consolidation of CFM (treatment × trial: $F_{(4,36)}$=4.371,

p=0.006; pairwise comparison between treatments at LTM2: $F_{(4,36)}$=8.246, p=0.002; DTZ: n=6, Veh: n=6). (**B**) There was no difference between the levels of freezing during CFC between the two groups (effects of treatment: $F_{(1,12)}$=2.362, p=0.15; trial: $F_{(1,12)}$=180.390, p<0.001; treatment × trial: $F_{(1,12)}$=0.878, p=0.367). DTZ infusion prior to LTM1 does not affect retrieval of the CFM at LTM1 or in subsequent trials (effect of treatment $F_{(1,12)}$=0.32, p=0.582, trial: $F_{(2,24)}$=5.035, p=0.015; treatment × trial interaction: $F_{(2,24)}$=1.305, p=0.290). DTZ: n=7; Veh: n=7. The effect of trial reflects the reduction in freezing response with multiple recalls indicative of normal between session extinction in the absence of DTZ. (**C**) DTZ infusion before pre-exposure blocks the establishment of latent inhibition of CFM (treatment × trial interaction: $F_{(4,40)}$=5.381, p=0.001; treatment comparisons at LTM1: $F_{(4,40)}$=10.364, p=0.009, LTM2: $F_{(4,40)}$=5.9178, p=0.035; DTZ: n=6, Veh: n=6). Data shown as means ±SEM. *p <0.05, **p<0.01 (two-way repeated measures ANOVA followed by Tukey adjustment of p-values for contrasts).

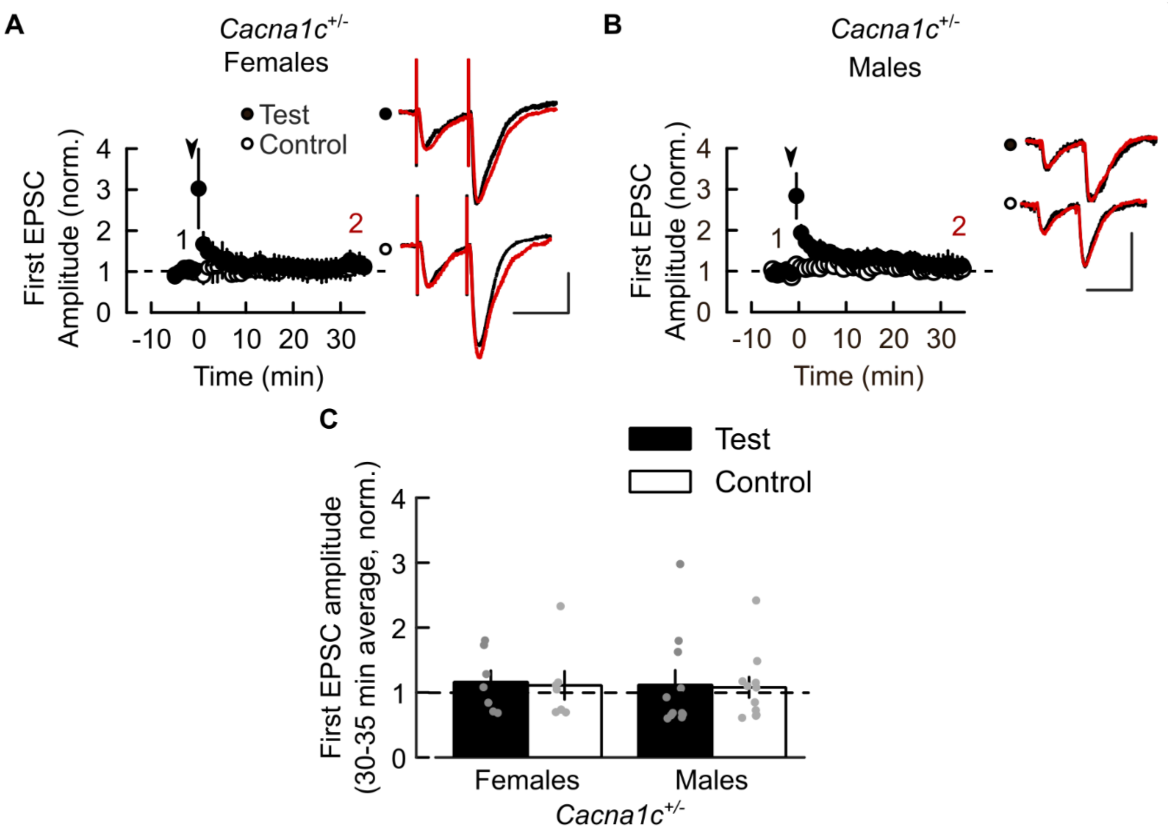

Figure S3. The deficit of TBP-induced SC-CA1 LTP is present in both male and female *Cacna1c*$^{+/-}$ rats. (**A**,**B**) Time course of EPSC amplitude normalized to 5 min before delivery of TBP protocol to the Test pathway (arrowheads). Insets: Average EPSC waveforms 5 min (1, black) and 30-35 min (2, red) after TBP. Scale bars: 50 pA, 50 ms. (**C**) Summary of normalized EPSC amplitude change 30-35 min after TBP. The deficit of SC LTP induction with TBP is present in both female (A) and male (B) *Cacna1c*$^{+/-}$ littermates (aggregate cohort data shown in Fig. 2B,D). Effect of sex: p=0.14, LR(1)=2.09; sex × pathway interaction: p=0.75, LR(1)=0.096; Test versus Control pathway contrasts, females: p=0.98, n=7(4); males: p=0.92, n=10(8). Sample sizes given as cells(animals). TBP protocol schematic is shown in Fig.2A.

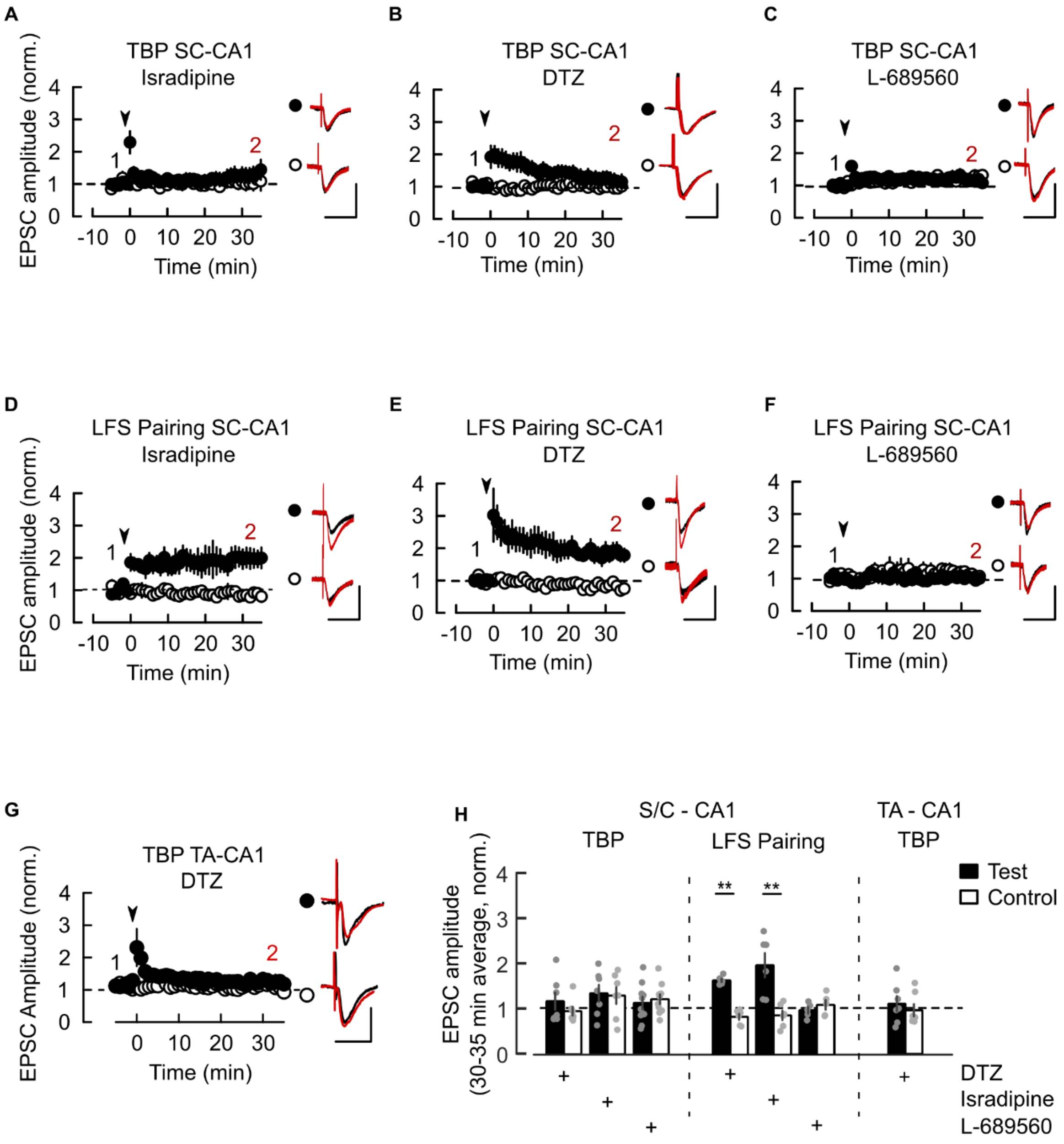

Figure S4. NMDAR and L-VGCC dependence of LTP at SC-CA1 and TA-CA1 synapses in *Cacna1c*$^{+/+}$ *ex vivo* slices. (**A-C**) TBP-induced LTP at SC-CA1 synapses is blocked by L-VGCC antagonists Isradipine (10 µM, A) or diltiazem (DTZ, 100 µM, B) and by the NMDAR antagonist L-689560 (5 µM, C). (**D-F**) SC-CA1 LTP induced by LFS-pairing is insensitive to L-VGCC antagonists Isradipine (D) or DTZ (E) but is blocked by L-689560 (F). (**G**) TBP-induced LTP at TA-CA1 synapses is blocked by DTZ. Plots in A-G: Time course of EPSC amplitude normalized to 5 min before delivery of TBP protocol to the Test pathway (arrowheads). *Insets*: Average EPSC waveforms 5 min (1, black) and 30-35 min (2, red) after TBP. Scale bars: 50 pA, 50 ms. (**H**) Summary of normalized EPSC

amplitude change 30-35 min after induction of LTP, in A-G. Pairwise Test vs Control for TBP-induced LTP at SC-CA1: Isradipine: $p=0.99$, $n=7(5)$; DTZ: $p=0.9$, $n=7(6)$; L-689560: $p=0.927$, $n=10(6)$; LFS-pairing LTP in SC-CA1, Isradipine: $p=0.0026$, $n=6(5)$; DTZ: $p=0.0013$, $n=6(5)$; L-689560: $p=0.9$, $n=5(4)$; TBP LTP at TA-CA1: DTZ: $p=0.86$, $n=7(5)$. Sample sizes given as cells(animals). All drugs were bath applied throughout the recording. Data shown as means ± SEM. *$p <0.05$, **$p<0.01$ (two-way ordinal regression and Tukey adjustment of p-values for contrasts).

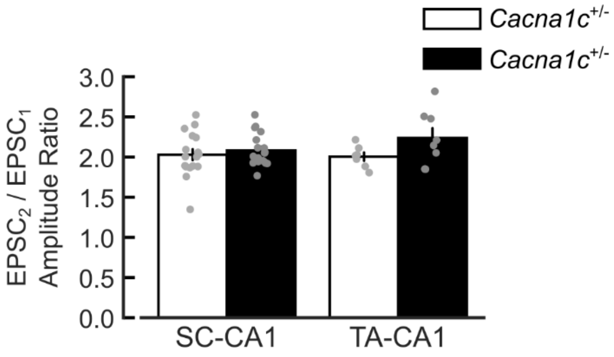

Figure S5. Paired-pulse facilitation shows no genotype effect on transmitter release at SC-CA1 and TA-CA1 synapses. Paired-pulse facilitation in slices from *Cacna1c*$^{+/-}$ and *Cacna1c*$^{+/+}$ animals before LTP induction using the TBP protocol, at SC-CA1 synapses (p = 0.5149, *Cacna1c*$^{+/-}$ n = 17(12); *Cacna1c*$^{+/+}$ n = 16(9), data related to experiments in Figure 2B,C) and TA-CA1 synapses (p = 0.107, *Cacna1c*$^{+/-}$ n = 8(7); *Cacna1c*$^{+/+}$ n = 7(5) related to experiments in Figure 2J,K), two sample t-tests. Data shown as means ± S.E.M. Sample sizes given as cells (animals).

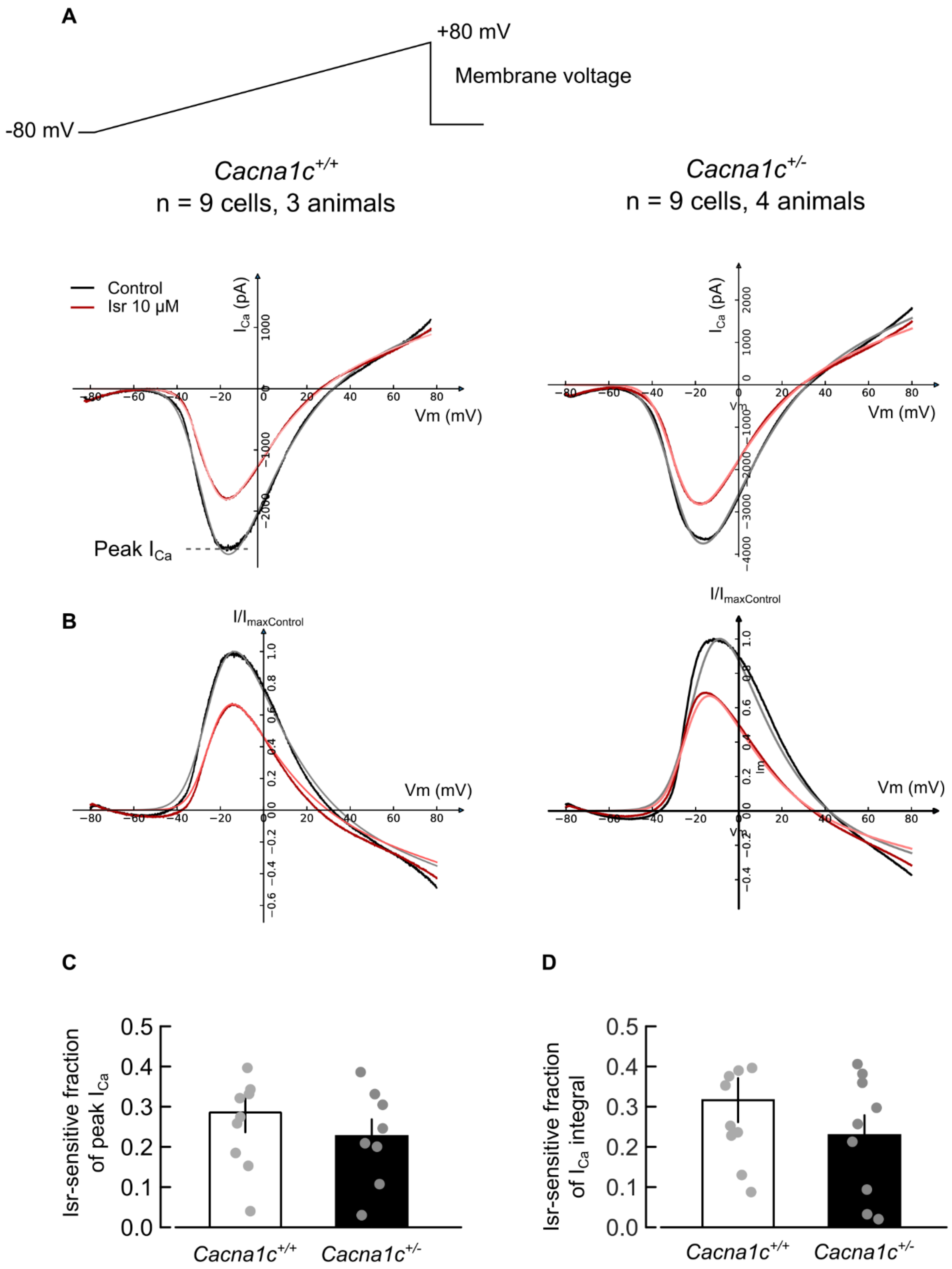

Figure S6. Isradipine-sensitive whole-cell Ca^{2+} currents in *Cacna1c*$^{+/-}$ vs *Cacna1c*$^{+/+}$ CA1 pyramidal neurons. (**A**) Example of current-voltage relationships for whole-cell ca^{2+} currents (I_{Ca}) during membrane voltage ramps as depicted in the top schematic and fitted with

the non-linear model described in (1). (**B**) Peak-normalized ($I/I_{max\ Control}$) current-voltage relationships in (A) obtained by normalizing I_{Ca} during Isr to the peak inward I_{Ca} in Control (indicated in A). (**C,D**) Isr-sensitive fraction of peak I_{Ca} (C: *Cacna1c*$^{+/+}$: 0.28 ± 0.05, *Cacna1c*$^{+/-}$: 0.22 ± 0.04, p=0.41;) and I_{Ca} integral (area under the curve between -40 and +25 mV, D: *Cacna1c*$^{+/+}$: 0.31 ± 0.06, *Cacna1c*$^{+/-}$: 0.23 ± 0.05, p=0.29). Statistical comparison: nested ANOVA with linear mixed effects model. Data represented as mean ± SEM.

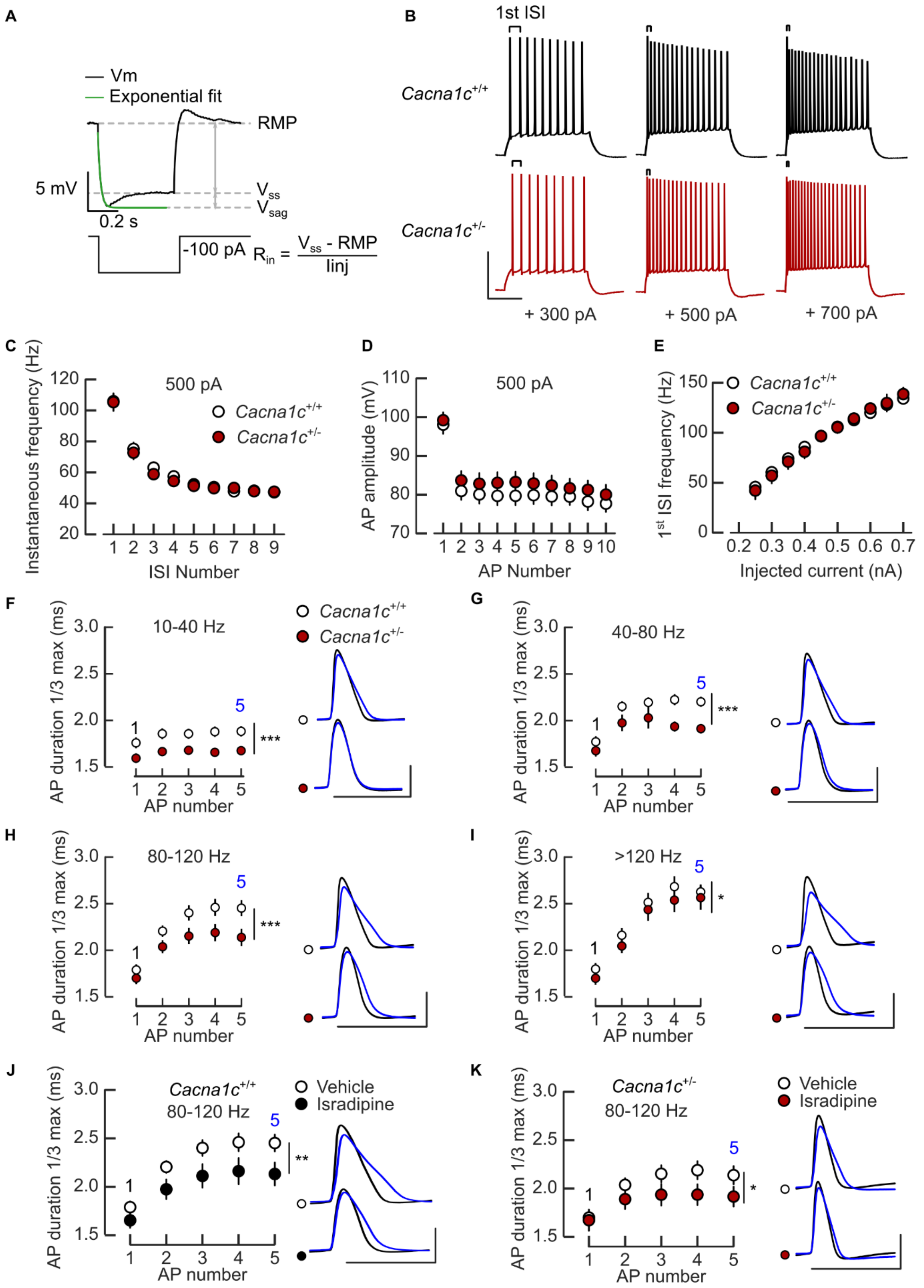

Figure S7. Reduced frequency-dependent broadening of somatic action potential waveforms in CA1 pyramidal neurons from *Cacna1c*$^{+/-}$ animals. (**A**) Schematic of passive

membrane properties measurement: resting membrane potential (RMP), input resistance (R_{in}), V_{sag} and the exponential fit for membrane time constant (τ_m) determination from membrane voltage recordings during hyperpolarizing current injections. (**B**) Examples of action potential (AP) trains discharged during 500 ms depolarising current (intensity shown underneath). ISI: inter-spike interval. (**C-E**) *Cacna1c*$^{+/+}$ and *Cacna1c*$^{+/-}$ neurons fire APs with comparable frequency adaptation (effect of spike number: $LR_{(9)}$=328.27, p<0.0001; genotype: $LR_{(1)}$=0.48, p=0.48, C), amplitude attenuation (effect of AP number: $LR_{(9)}$=430.12, p<0.0001; genotype: $LR_{(1)}$=0.11, p=0.73, D) and similar frequency – current intensity relationship (E). Data in C,D: first 10 AP fired during +500 pA current (500 ms); *Cacna1c*$^{+/+}$: n=43(15); *Cacana1c*$^{+/-}$: n=30(10); sample sizes given as cells(animals). (**F-I**) Frequency-dependent broadening of somatic APs is attenuated in *Cacna1c*$^{+/-}$ *versus Cacna1c*$^{+/+}$ neurons. Genotype effect at 10-40 Hz (F): $LR_{(1)}$=30.67 p<0.0001; 40-80 Hz (G): $LR_{(1)}$=41.12, p< 0.0001; 80-120 Hz (H): $LR_{(1)}$=22.83, p<0.0001; >120 Hz (I): $LR_{(1)}$ = 5.8, p=0.015. Sample sizes given as cell(animals) for each frequency band respectively, for *Cacna1c*$^{+/+}$ and *Canca1c*$^{+/-}$, at 10-40 Hz: n=36(15) and n=26(10); 40-80 Hz: n=30(15) and n=29(10); 80-120 Hz: n=43(15) and n=28(10); >120 Hz: n=37(15) and n=26(10). (**J,K**) Isradipine (10 µM) inhibits AP broadening at 80-120 Hz in both *Cacna1c*$^{+/+}$ ($LR_{(1)}$ = 7.16, p=0.007, n=26(11), J) and *Cacna1c*$^{+/-}$ ($LR_{(1)}$=5.63, p=0.017, n=13(3), K) neurons. Plots in F-K: AP durations for the first 5 AP. Insets: 1st (2, black) and 5th (5, blue) AP waveform; scale bars: 5 ms, 50 mV. When used, Isradipine was bath applied throughout the experiment. Data shown as means ±SEM. *p <0.05, **p < 0.01 and ***p < 0.001 (two-way ordinal regression with repeated measures and Tukey adjustment of p-values for contrasts).

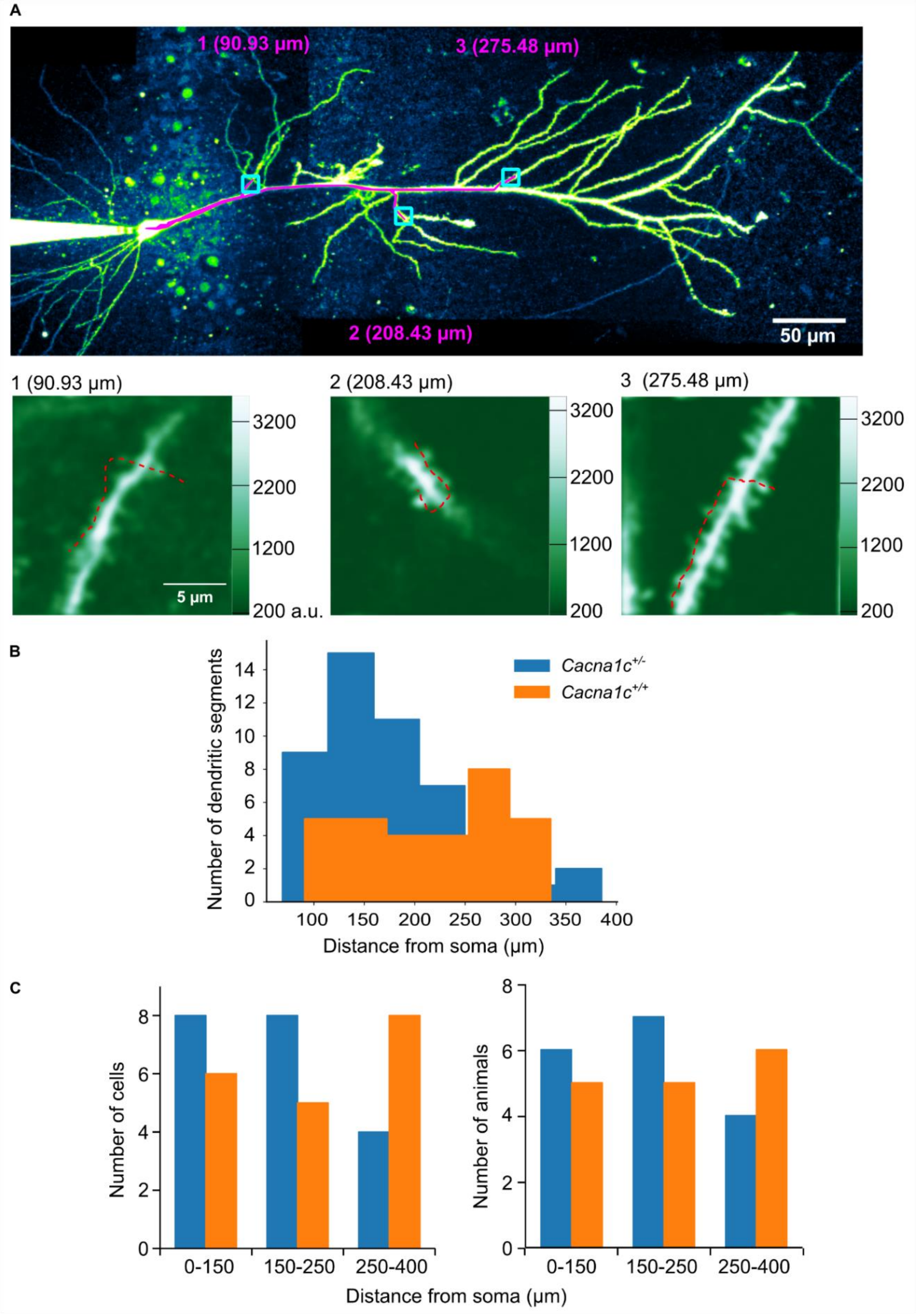

Figure S8. Location of spines imaged for calcium transients triggered by somatic action potentials. (**A**) *Top*: Z-projected image of a CA1 pyramidal neuron (AlexaFluor 594 channel) with the overlaid "paths" (pink) used to measure the distance from the soma to three imaging

locations (blue squares). The “paths” were traced and measured using the Single Neurite Tracer (Image/J). *Bottom*: Raster scans through the imaging locations shown in the Z-projected image (trajectory of line scan overlaid in red); a.u.: arbitrary units. Imaging was performed on spines on a single dendritic segment at a given location. Imaging locations were selected pseudo-randomly, ensuring that the imaged spines were located on distinct dendritic branches originating from the main trunk at least 30 µm apart. (**B**) Distribution of soma-spine distances. Spines on the same dendritic segment were attributed the same distance from the soma. (**C**) Distribution of the somatic distance zones across cells (left) and animals (right) in both genotypes.

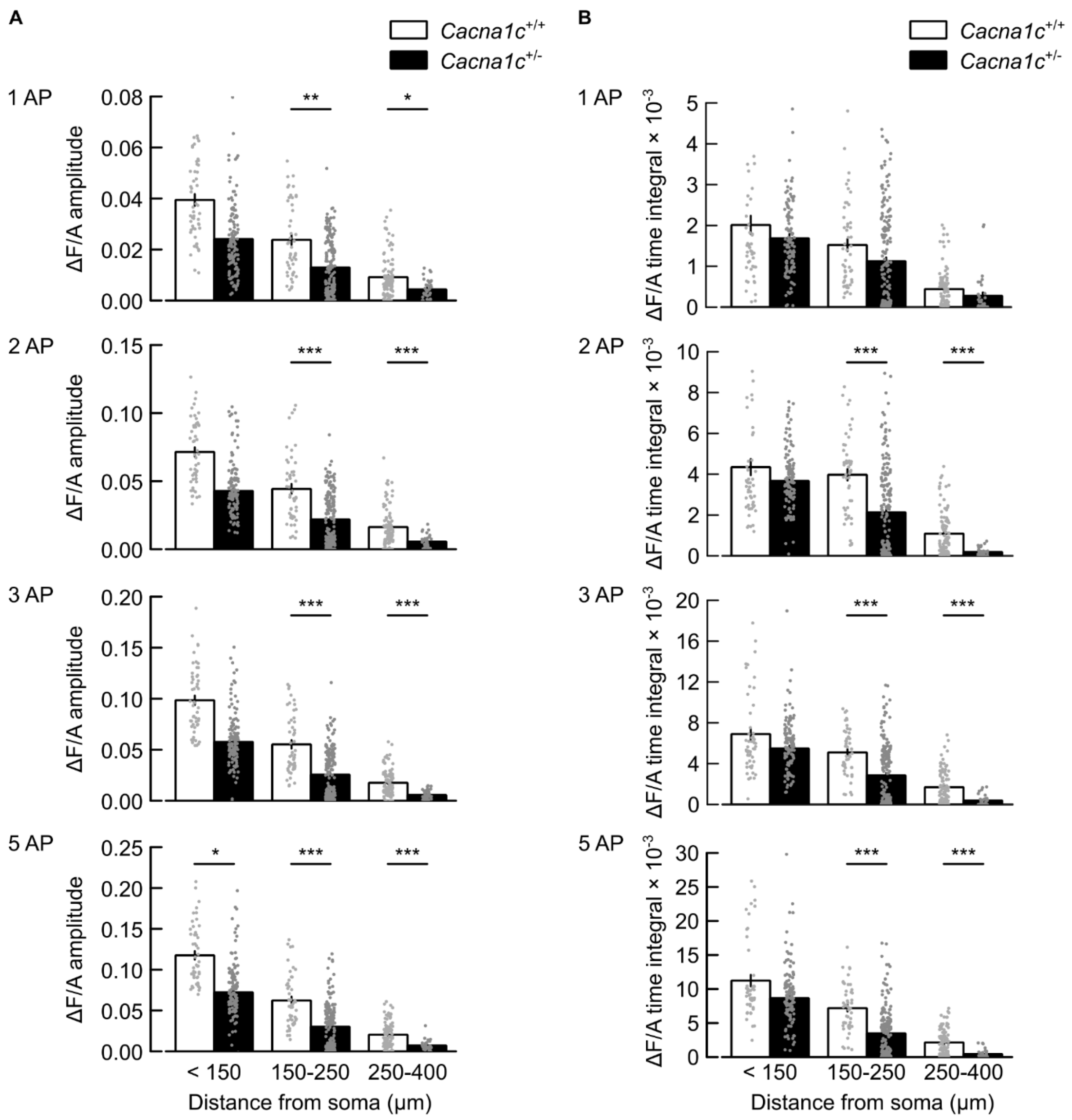

Figure S9. Attenuation of action potential-triggered spine calcium transients with distance from soma in CA1 pyramidal neurons. (**A,B**) Summary of peak ΔF/A amplitude (A) or ΔF/A time integral (B) of spine Ca^{2+} transients (APCaTs) evoked by 1, 2, 3 or 5 back-propagated action potentials (AP, 100 Hz intra-burst, top to bottom) *versus* spine distance from soma. APCaTs evoked with bursts of two to five APs are significantly smaller in spines located beyond 150 µm from soma, in *Cacna1c*$^{+/-}$ compared to *Cacna1c*$^{+/+}$ neurons. Statistical analysis results and sample sizes are given in Supplementary Table S2 and Figure S7; all p-values for pairwise comparisons are given in Supplementary Table S3. Data for the 5 AP APCaTs are reproduced from Fig. 3D,E. Data shown as means ±SEM. *p <0.05 and **p < 0.01 (two-way repeated measures ANOVA followed by Tukey adjustment of p-values for contrasts).

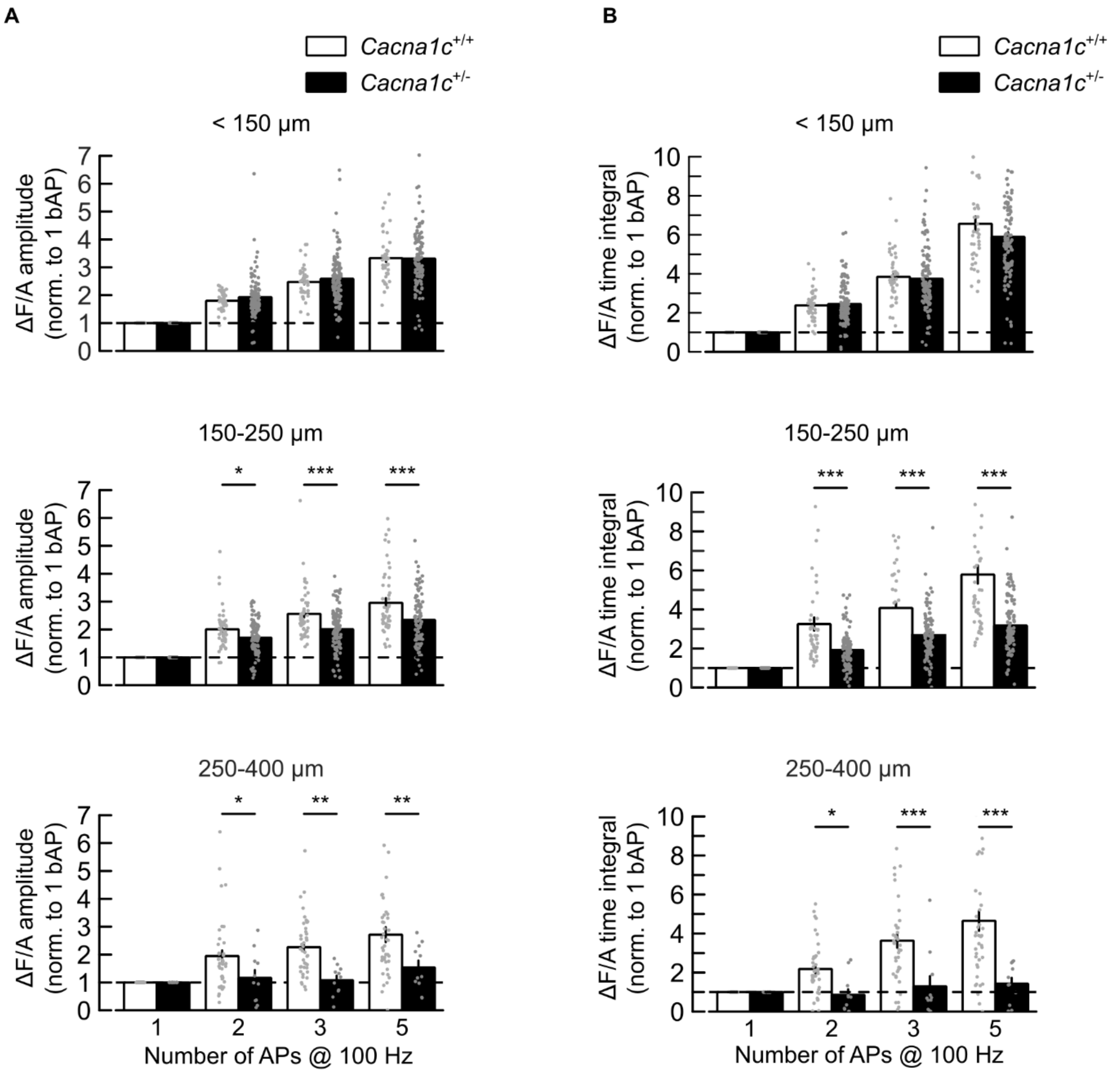

Figure S10. Summation of action potential-triggered spine calcium transients with the number of action potentials. (**A,B**) Summary of peak ΔF/A amplitude (A) or ΔF/A time integral (B) of spine Ca^{2+} transients (APCaTs) evoked by 1, 2, 3 or 5 back-propagated action potentials (AP, 100 Hz intra-burst) normalized to the values at 1 AP, in the three distance zones analysed (top-to-bottom). In spines located beyond 150 µm from the soma, the summation of APCaTs with the number of APs is reduced in *Cacna1c*$^{+/-}$ *versus Cacna1c*$^{+/+}$ neurons. Statistical analysis results and sample sizes are given in Supplementary Table S2 and Figure S7; all p-values for pairwise comparisons are given in Supplementary Table S3. Middle panels (150-250 µm) are reproduced from Fig. 3F,G. Data shown as means ±SEM. *p <0.05, **p < 0.01 and ***p<0.001 (two-way repeated measures ANOVA followed by Tukey adjustment of p-values for contrasts).

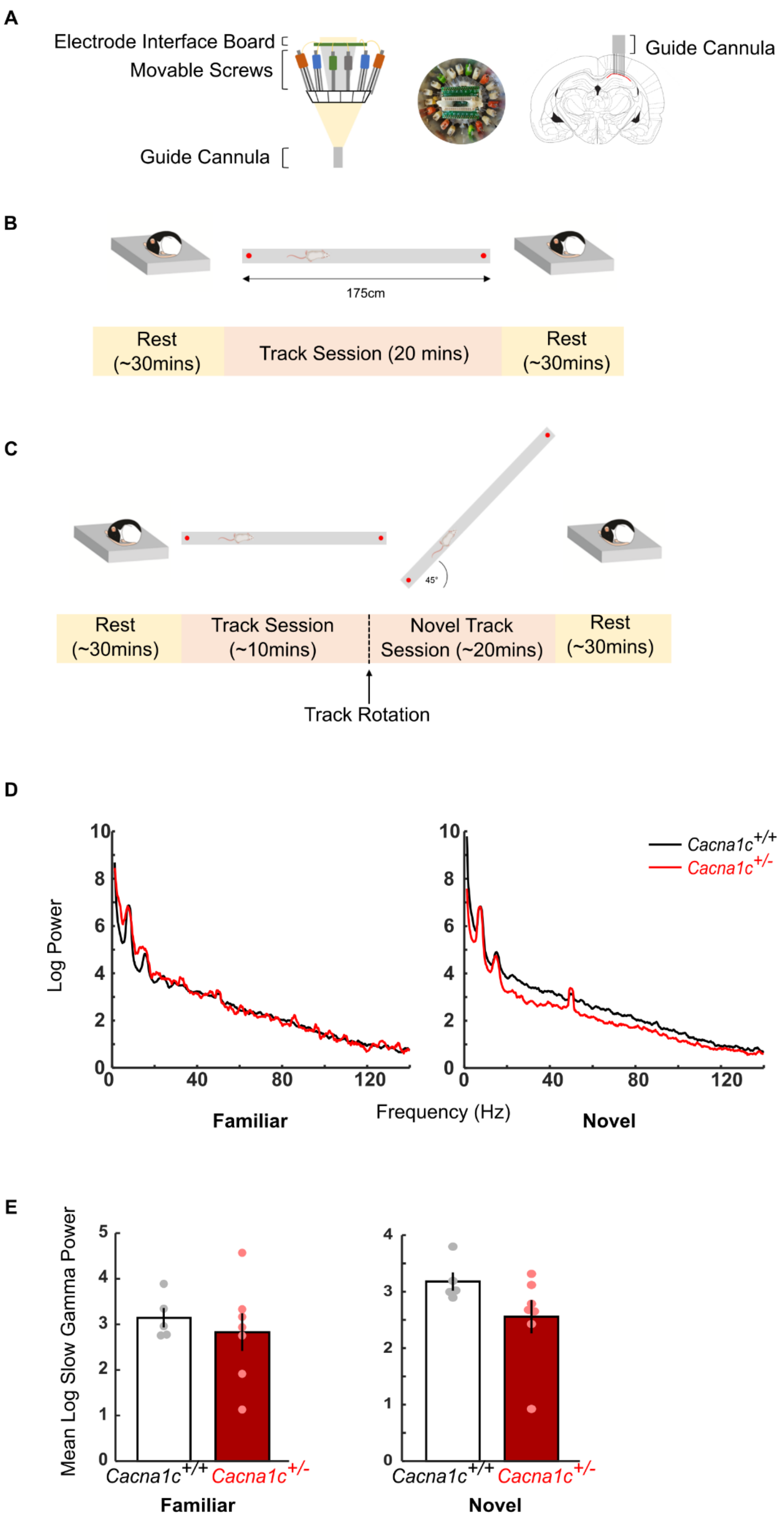

Figure S11. Local field potential recordings in dorsal CA1 during novel vs familiar track behavioural protocol. (**A**) Tetrode Microdrive used for recording in vivo local field potentials. Left: Schematic of Microdrive viewed from the side. Middle: Photo of Microdrive

viewed from the top. Right: Position of guide cannula during surgery and targeting of tetrodes to dorsal CA1 of hippocampus. Red line indicates dorsal CA1 cell layer. (**B**) Familiar track behavioural protocol. Following a ~30-minute rest period, rats freely run back and forth on the linear track for sucrose rewards followed by another ~30-minute rest period. (**C**) Novel Track Behavioural Protocol. As (B) but midway through the track session, rats were taken off the track and the track was rotated 45° before being placed back on to continue running freely for sucrose rewards. (**D**) Power spectra taken from track runs on the familiar (left) and novel track (right). (**E**) Mean slow gamma (25-55Hz) power did not differ on the familiar ($p = 0.5607$; Student's t-test) or novel track ($p = 0.1306$; Student's t-test). (*Cacna1c*$^{+/+}$: n = 5; *Cacna1c*$^{+/-}$: n=7; Data was obtained in the same cohort as in Fig. 4 and is presented as mean ± SEM).

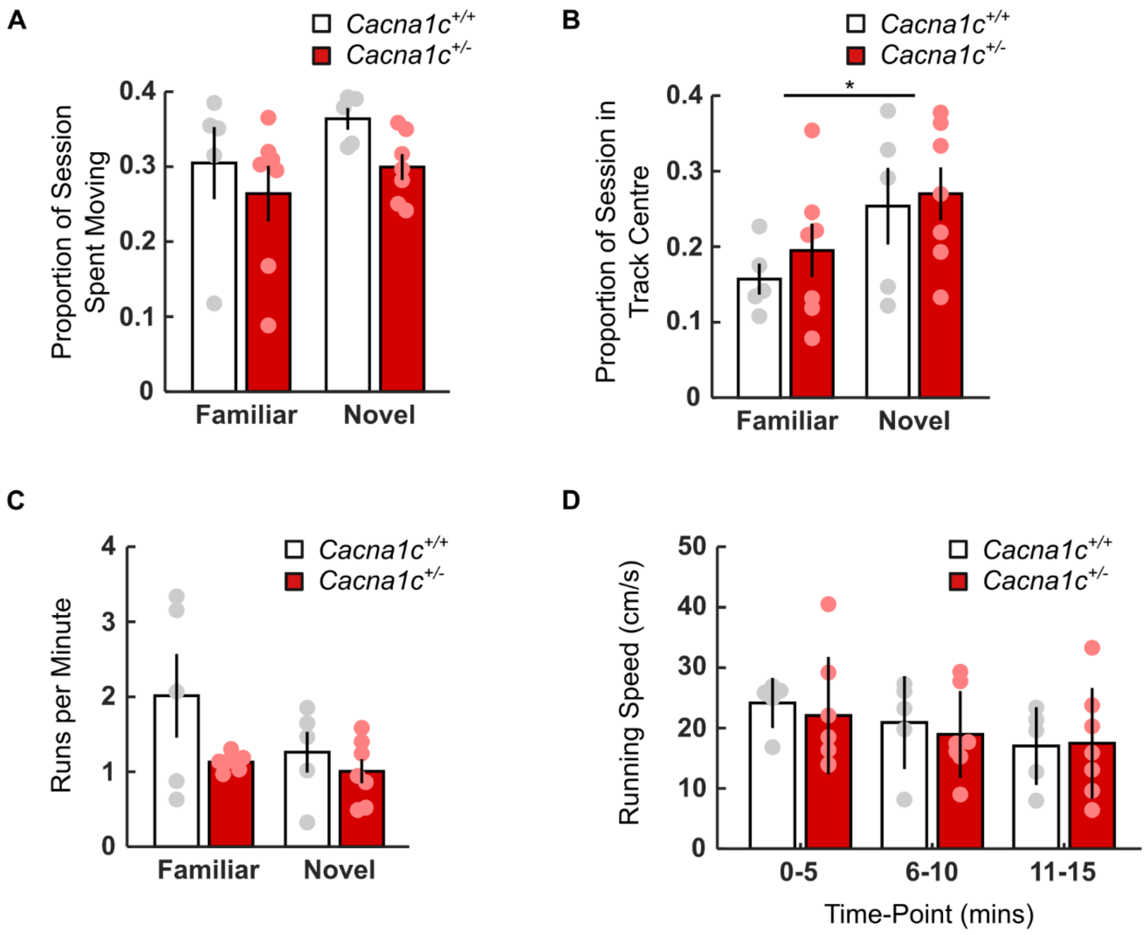

Figure S12. Behaviour on the familiar and novel linear track. (A) Proportion of familiar and novel track session spent moving compared to stationary. No significant effect of genotype ($F_{(1,20)}$=2.66, p=0.119), session ($F_{(1,20)}$=2.15, p=0.158) or genotype x session interaction ($F_{(1,20)}$=0.13, p=0.718). **(B)** Proportion of familiar and novel track session spent in the track centre (inner 75% of track area) vs. track periphery (outer 25% of track area). This analysis revealed a significant effect of session ($F_{(1,20)}$=5.22, p=0.033) though no significant effect of genotype ($F_{(1,20)}$=0.53, p=0.477), and no genotype x session interaction ($F_{(1,20)}$=0.08, p=0.777). **(C)** Mean number of runs per minute of recording during the familiar and novel track session. No significant effect of genotype ($F_{(1,20)}$=4.25, p=0.052), session ($F_{(1,20)}$=2.5, p=0.129) or genotype x session interaction ($F_{(1,20)}$=1.29, p=0.269). **(D)** Mean running speed during 5-minute bins across the novel track session. This analysis revealed a significant effect of time ($F_{(2,20)}$=3.84, p=0.039) though no effect of genotype ($F_{(2,20)}$=0.23, p=0.798). Group sizes: *Cacna1c*$^{+/+}$: n=5; *Cacna1c*$^{+/-}$: n=7; All data presented as mean ± SEM. *p <0.05, Two-way ANOVA (A-C) and two-way repeated measures ANOVA (D)

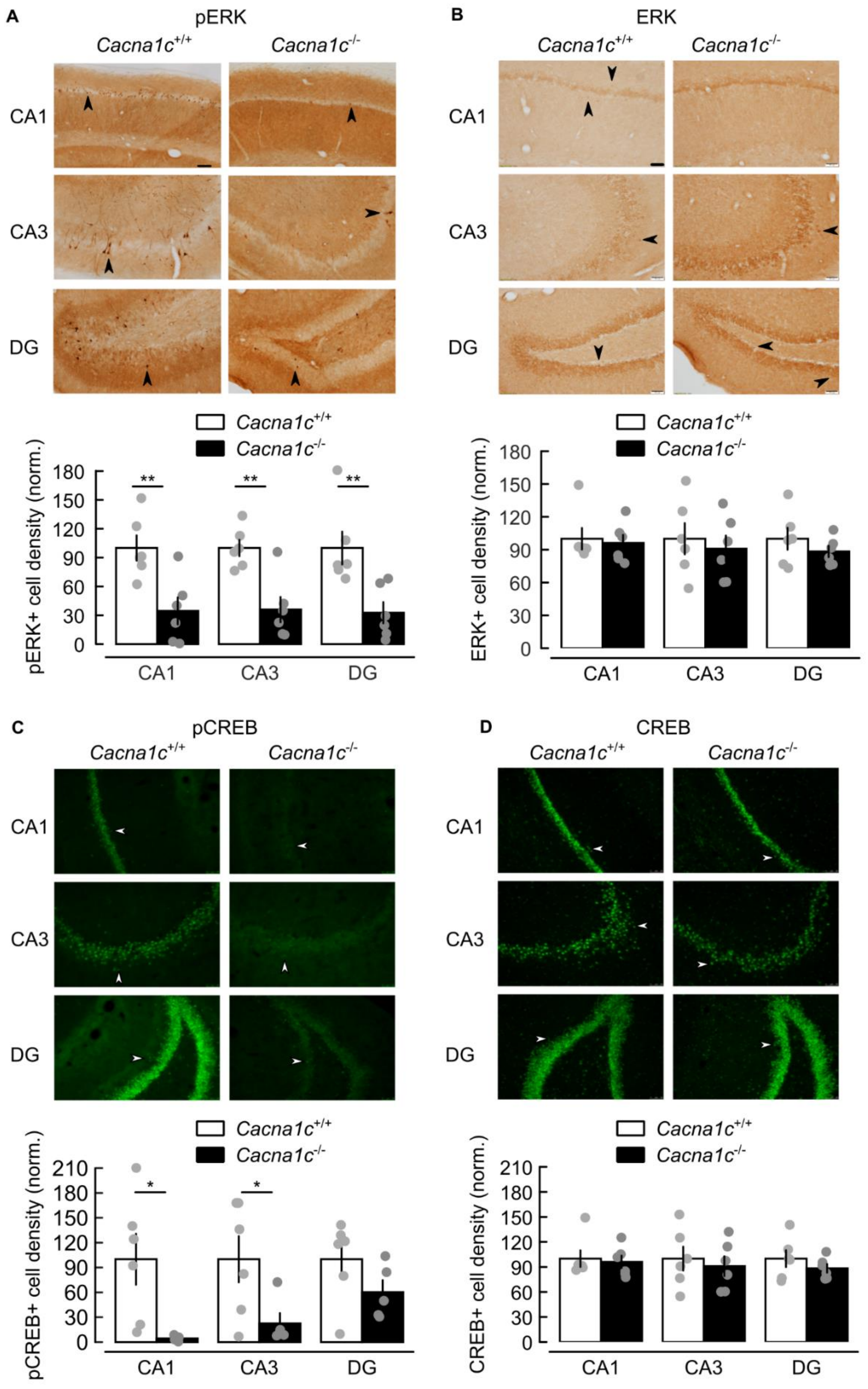

Figure S13. Reduced basal levels of phosphorylated ERK and phosphorylated CREB in the hippocampus of *Cacna1c*$^{+/-}$ rats. (**A**) Baseline levels of pERK are reduced in the hippocampus of *Cacna1c*$^{+/-}$ compared to *Cacna1c*$^{+/+}$ rats. Effects of genotype:

$F_{(1,10)}$=14.617, p=0.003 and region: $F_{(2,20)}$=0.038, p=0.962; genotype × region interaction: $F_{(2,20)}$=0.038, p=0.962; independent sample t-tests for pERK-positive cell density in CA1: $t_{(10)}$=3.423, p=0.007; CA3: $t_{(10)}$ = 4.095, p=0.002; DG: $t_{(10)}$=3.311, p=0.008. (**B**) Similar levels of total ERK expression in both genotypes (F < 1). (**C**) Reduced baseline hippocampal levels of pCREB in *Cacna1c*$^{+/-}$ compared to *Cacna1c*$^{+/+}$ rats. Effects of genotype: $F_{(1,9)}$=6.719, p=0.029 and region: $F_{(2,18)}$=2.524, p=0.108; genotype × region: $F_{(2,18)}$=2.524, p=0.108; independent sample t-tests for pCREB-positive cell density in CA1: $t_{(5.019)}$=3.105, p=0.027; CA3: t(6.875) = 2.537, p = 0.039; DG: $t_{(10)}$=1.549, p=0.156. (**D**) Levels of total CREB expression are similar in both genotypes (F < 1). **All panels**: *Top*: examples of immunoperoxidase (A,B) and immunofluorescence (C,D) staining; arrow heads indicate immunopositive cells. *Bottom*: summary of immunopositive cell densities (mean ± SEM), in the dorsal hippocampal regions CA1, CA2 and dentate gyrus (DG), normalized to the average value in *Cacna1c*$^{+/+}$ tissue. The data was obtained in a cohort separate from that of Figure 5A,B; sample sizes: *Cacna1c*$^{+/+}$ (n = 6) and *Cacna1c*$^{+/-}$ (n=6) animals. Scale bars: immunoperoxidase images: 100 µm; immunofluorescence: 50 µm. Data shown as means ± SEM. *p <0.05 and **p < 0.01, two-way repeated measures ANOVA followed by independent sample t-test.

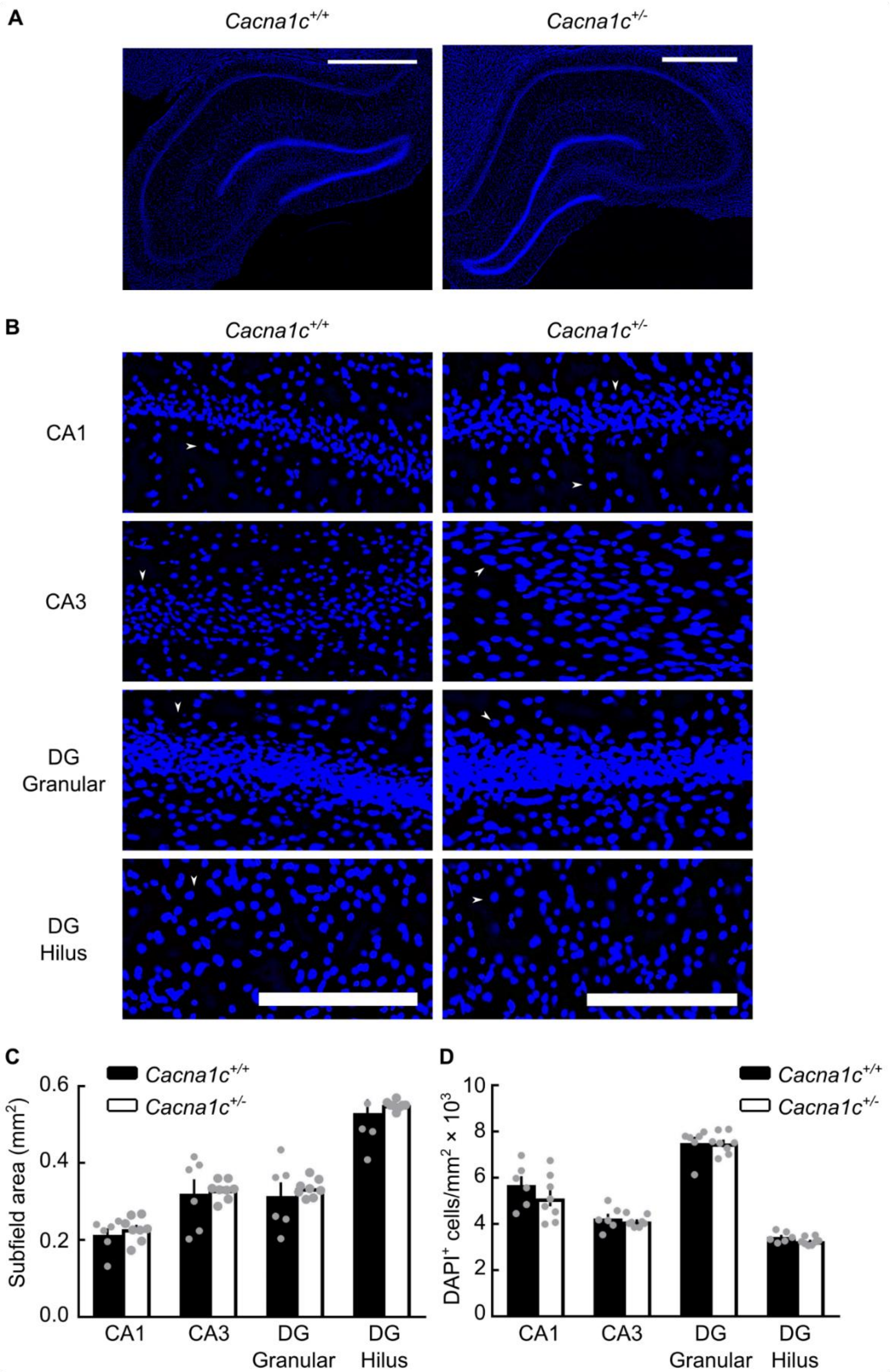

Figure S14. ***Cacna1c*$^{+/-}$ and *Cacna1c*$^{+/+}$ animals have similar hippocampal morphology and cellular densities in hippocampal subfields.** (**A,B**) DAPI fluorescence images of whole hippocampi (A) and hippocampal subfields (B) form both genotypes. Arrowheads in (B) indicate DAPI-stained cell nuclei. Scale bars: 1 mm (A) and 200 µm (B). (**C**) No significant

genotype effect on the areas of hippocampal subfields. CA1: $F(1, 13) = 0.612$, $p = 0.449$; CA3: $F(1, 13) = 0.088$, $p = 0.772$; dentate gyrus granular cell layer (DG Granular): $F(1, 13) = 0.404$, $p = 0.537$; DG Hilus: $F(1, 13) = 0.455$, $p = 0.513$. (**D**). No significant genotype effect on cell densities in hippocampal subfields. CA1: $F(1, 13) = 1.227$, $p = 0.290$; CA3: $F(1, 13) = 0.360$, $p = 0.559$; DG Granular: $F(1, 13) = 0.002$, $p = 0.964$; DG Hilus: $F(1, 13) = 2.870$, $p = 0.116$. (C, D): Two-way ANOVA. Data represented as mean ± SEM.

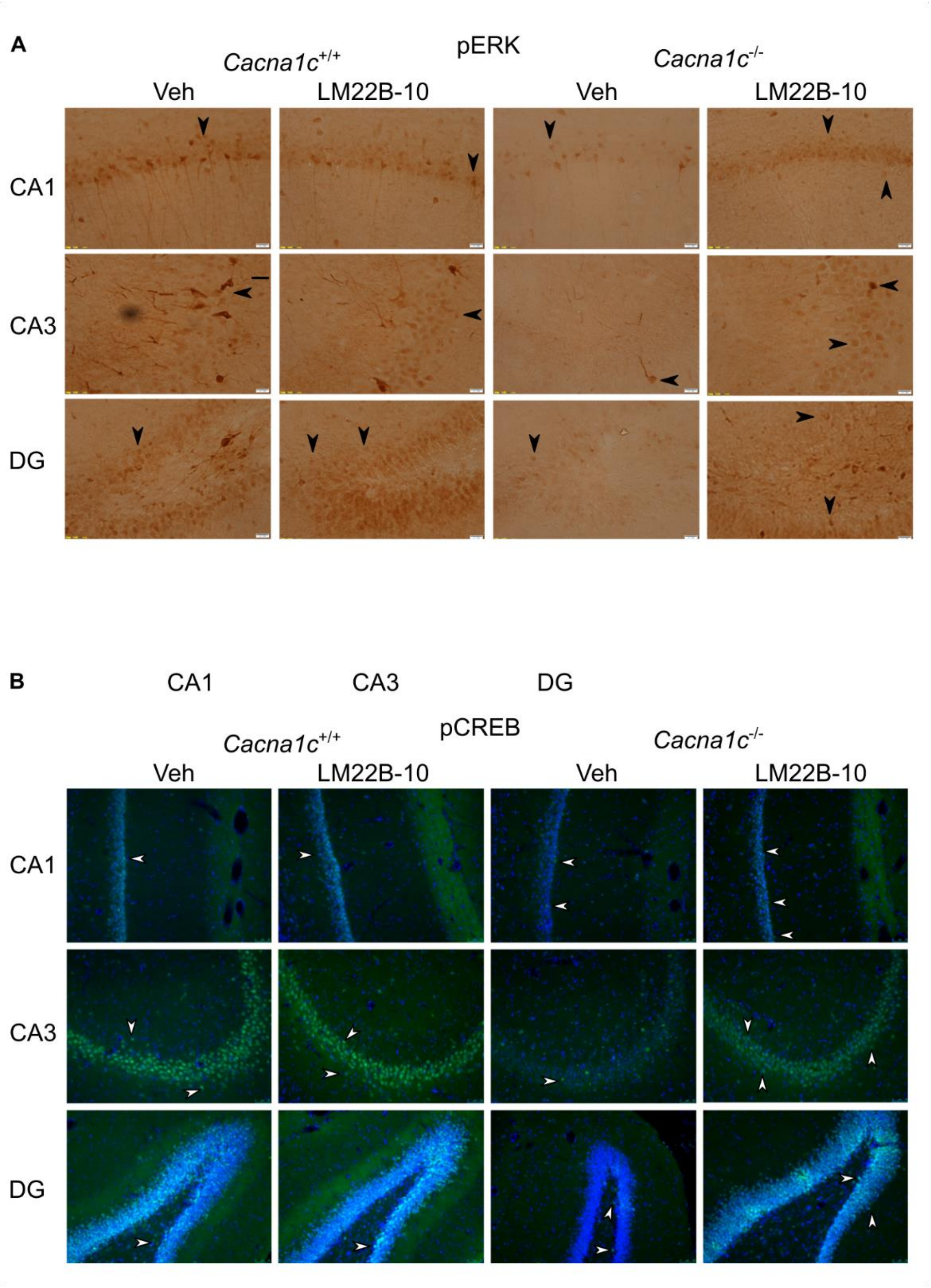

Figure S15. Rescue of basal levels of phosphorylated ERK and phosphorylated CREB in the dorsal hippocampus of *Cacna1c*$^{+/-}$ rats with systemic administration of LM22B-10 (related to Figure 5A,B). (**A,B**) Examples of microscopy sections with pERK immunoperoxidase (A, dark brown) and pCREB immunofluorescence (B, green) for data in Figure 5. Sections in (B) were counterstained for cellular nuclei with DAPI (blue). Arrow heads indicate immunopositive cells.

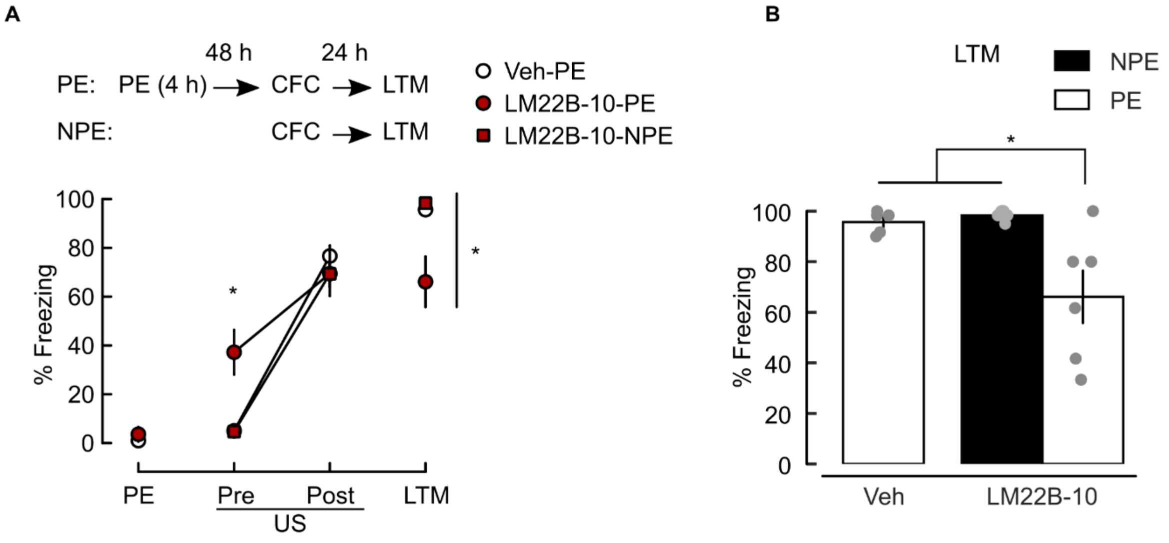

Figure S16. Systemic administration of LM22B-10 rescues LI of CFC in *Cacna1c*$^{+/-}$ animals. (**A**)Top schematic: paradigm sessions, with notations as in Fig. S1. Animals received an i.p. bolus of either LM22B-10 (25 mg/kg; pre-exposed: LM22B-10-PE, n=6; non pre-exposed: LM22B-10-NPE, n=6) or vehicle (pre-exposed: Veh-PE, n=5) 60 min before each stage. This schedule was chosen to avoid effects of state-dependency. Significantly different freezing behaviour in LM22B-10-PE *versus* Veh-PE animals (treatment × trial interaction: $F_{(3,24)}$=11.653, p<.0001). Freezing behaviour at Pre-US trial is stronger in LM22B-10-PE animals *versus* Veh-PE ($F_{(3,24)}$=7.858, p=0.023). (**B**) Freezing response at LTM is significantly smaller in LM22B-10-PE *versus* Veh-PE animals ($F_{(3,24)}$=5.638, p=0.045) and *versus* LM22B-10-NPE animals ($t_{(5.0582)}$=3.099, p=0.027). Data shown as means ±SEM. *p <0.05, determined by univariate ANOVA and independent sample t-test.

Table S1. Passive membrane and action potential firing properties of CA1 pyramidal neurons in *Cacna1c*$^{+/+}$ and *Cacna1c*$^{+/-}$ littermates.

**Passive membrane properties:**	***Cacna1c*$^{+/+}$**	***Cacna1c*$^{+/-}$**	***p*† (U)**
RMP (mV)	-71.77 ± 0.35	-70.99 ± 0.45	0.39 (310)
R_{in} (MΩ)	66.57 ± 3.46	62.0 ± 3.22	0.6 (345)
τ_M (ms)	20.3 ± 1.3	23 ± 1.5	0.15 (337)
% Sag	2.05 ± 0.17	1.94 ± 0.11	0.85 (261)
% Rebound	10.35 ± 0.52	9.82 ± 0.39	0.6 (245)
**Action potential (AP) firing properties:**			
I_{rh} (pA)	201.55 ± 11.36	237.55 ± 17.13	0.12 (511)
Number of AP‡	18.99 ± 0.79	16.72 ± 1.22	0.23 (751)
Firing threshold (mV)‡	-54 ± 1.07	-54.55 ± 0.77	0.57 (595)
First AP amplitude (mV)‡	99.99 ± 1.48	99.22 ± 1.31	0.42 (693)
First AP maximum dV/dt (V/s)‡	390.31 ± 14.24	400.27 ± 14.01	0.54 (591)

† p-value and U statistic, two-sided Mann-Whitney U test, *Cacna1c*$^{+/+}$: n=43 cells, 15 animals; *Cacna1c*$^{+/-}$: n=30 cells, 10 animals.

‡ measured at 500 pA current injection.

Table S2: **Statistical comparisons of APCaTs by regions of distance from soma.** File Tigaret et al Table S2.xlsx

Table S3: P-values for pairwise comparisons of APCaTs in Cacna1c$^{+/+}$ vs Cacna1c$^{+/-}$ CA1 pyramidal neurons. File Tigaret et al Table S3.xlsx
